# Transcranial alternating current stimulation entrains alpha oscillations by preferential phase synchronization of fast-spiking cortical neurons to stimulation waveform

**DOI:** 10.1038/s41467-021-23021-2

**Published:** 2021-05-25

**Authors:** Wei A. Huang, Iain M. Stitt, Ehsan Negahbani, D. J. Passey, Sangtae Ahn, Marshall Davey, Moritz Dannhauer, Thien T. Doan, Anna C. Hoover, Angel V. Peterchev, Susanne Radtke-Schuller, Flavio Fröhlich

**Affiliations:** 1 Department of Psychiatry, University of North Carolina, Chapel Hill, NC USA; 2 Carolina Center for Neurostimulation, University of North Carolina, Chapel Hill, NC USA; 3 Neuroscience Center, University of North Carolina, Chapel Hill, NC USA; 4 Department of Mathematics, University of North Carolina, Chapel Hill, NC USA; 5 School of Electronic and Electrical Engineering, Kyungpook National University, Daegu, South Korea; 6 Department of Psychiatry and Behavioral Science, Duke University, Durham, NC USA; 7 Department of Biomedical Engineering, Duke University, Durham, NC USA; 8 Department of Electrical and Computer Engineering, Duke University, Durham, NC USA; 9 Department of Neurosurgery, Duke University, Durham, NC USA; 10 Department of Cell Biology and Physiology, University of North Carolina, Chapel Hill, NC USA; 11 Department of Biomedical Engineering, University of North Carolina, Chapel Hill, NC USA; 12 Department of Neurology, University of North Carolina, Chapel Hill, NC USA

**Keywords:** Neuroscience, Computational neuroscience

## Abstract

Computational modeling and human studies suggest that transcranial alternating current stimulation (tACS) modulates alpha oscillations by entrainment. Yet, a direct examination of how tACS interacts with neuronal spiking activity that gives rise to the alpha oscillation in the thalamo-cortical system has been lacking. Here, we demonstrate how tACS entrains endogenous alpha oscillations in head-fixed awake ferrets. We first show that endogenous alpha oscillations in the posterior parietal cortex drive the primary visual cortex and the higher-order visual thalamus. Spike-field coherence is largest for the alpha frequency band, and presumed fast-spiking inhibitory interneurons exhibit strongest coupling to this oscillation. We then apply alpha-tACS that results in a field strength comparable to what is commonly used in humans (<0.5 mV/mm). Both in these ferret experiments and in a computational model of the thalamo-cortical system, tACS entrains alpha oscillations by following the theoretically predicted Arnold tongue. Intriguingly, the fast-spiking inhibitory interneurons exhibit a stronger entrainment response to tACS in both the ferret experiments and the computational model, likely due to their stronger endogenous coupling to the alpha oscillation. Our findings demonstrate the in vivo mechanism of action for the modulation of the alpha oscillation by tACS.

## Abstract (web summary)

Transcranial alternating current stimulation (tACS) can modulate cortical oscillations and associated long-lasting cognitive and behavioral functions in humans. Here, the authors provide in vivo evidence in ferrets on the mechanism of how weak electric fields in tACS can entrain neuronal activity.

## Introduction

Transcranial electric stimulation (tES) is a non-invasive brain stimulation modality that delivers a weak electric current of typically up to 2 mA zero-to-peak amplitude to the scalp^1^. Transcranial direct current stimulation (tDCS) and transcranial alternating current stimulation (tACS) are the two most common types of tES, where a constant or sinusoidal current waveform is used for stimulation, respectively. The majority of the stimulation current is shunted by the scalp^2^, so a relatively small fraction of the current actually enters the brain and produces electric fields in the range of 0.2–0.5 mV/mm^2–5^. Recently, controversy has engulfed the field due to the heterogeneity of behavioral findings^6–8^ and the claim that weak perturbations are not strong enough to entrain networks^8–11^.

Targeted tACS modulation of cortical oscillations and associated cognitive and behavioral functions have been demonstrated in a number of human studies^12–20^. In addition, clinical trials of tACS for the treatment of schizophrenia^21^, chronic pain^22^, and major depressive disorder^23^ have been reported. These clinical trials demonstrated successful target engagement and modulated alpha oscillations after repeated application of tACS. More is known about these “offline” (i.e., after stimulation) effects since tACS introduces non-trivial artifacts when used with non-invasive electrophysiology techniques such as EEG (electroencephalography)^24^. At the level of large-scale neuronal populations, animal studies have provided evidence for “online” (i.e., during stimulation) modulation of neural oscillators by tACS in vitro and in vivo^25–31^. At the single neuron level, tACS polarizes neurons with alternating polarity resulting in a subthreshold resonance where the hyperpolarization-activated cation current plays a key role in the neural response^32,33^. Despite these successful demonstrations that weak periodic electric fields interact with neural oscillators, the mechanism of action remains unclear. Using a computational model of a large-scale cortical network of spiking neurons, we previously studied how tACS entrains ongoing oscillations^31^. We found a triangular region of high synchrony between the stimulation waveform and endogenous neuronal oscillations where higher stimulation amplitudes yielded a wider range of entrained frequencies. This triangular region of stimulation amplitude and frequency pairs is referred to as an Arnold tongue, which is centered on the frequency of the endogenous oscillation. The Arnold tongue is typically found when studying the dynamical properties of coupled oscillators and when characterizing the regions in parameter space where phase-locking occurs between two oscillators^34^. These model-driven predictions along with the existing entrainment hypothesis, which is supported by in vitro studies and in vivo studies in anesthetized rodents, comprise the current mechanistic understanding of tACS. Despite the successful demonstration of long-lasting tACS effects in human studies, a full mapping of the space of stimulation parameters that demonstrates a pattern of synchronization in the form of the Arnold tongue remains missing. The major roadblocks for this gap are the technical challenges of simultaneous tACS and EEG recording in human studies, even with some recent attempts to avoid or filter the large electrical artifact of tACS^24,35–39^.

To address this gap in knowledge, we investigated neural entrainment of alpha oscillations by tACS in awake head-fixed ferrets. We used tACS parameters that induced (computationally estimated) electric field amplitudes of <0.5 mV/mm, which are comparable to the estimated field amplitudes typically reported for human tACS paradigms. We first demonstrated the presence of alpha oscillations (11–17 Hz) in the awake head-fixed ferret with simultaneous recording from the posterior parietal cortex (PPC), the primary visual cortex (VC), and the lateral posterior nucleus/Pulvinar complex of thalamus (LP). In functional agreement with human and non-human primate studies of alpha oscillations, the recordings pointed to the top-down control of the network by alpha oscillations^40,41^. The alpha amplitude was strongest in PPC, which influenced both VC and LP regions in the alpha-frequency band as demonstrated by functional and effective connectivity between these areas. Further analysis on putatively classified neuron types showed that the alpha synchrony between the network oscillations and neuronal firing rates was stronger for narrow-spiking neurons compared to broad-spiking neurons. To probe for the presence of an Arnold tongue, the hypothesized mechanism of action of tACS, we systematically varied both stimulation amplitude and frequency. We found evidence supportive of the Arnold tongue for PPC neurons, and narrow-spiking neurons showed stronger phase-locking to tACS when compared to broad-spiking neurons. Despite the comparable amplitude of tACS electric field in all three studied regions, we did not find a clear Arnold tongue for VC or LP neurons. In addition, we addressed and excluded several potential confounds such as entrainment by peripheral stimulation. We finally examined the effect of tACS in a computational model of the thalamo-cortical network, which confirmed the main features of our experimental findings. Our work provides in vivo evidences on how weak electric fields (<0.5 mV/mm) can entrain neuronal activity and supports the model-driven predictions of the Arnold tongue as the mechanism of action of tACS.

## Results

### Endogenous alpha-band oscillations in awake head-fixed ferrets

**Fig. 1:**
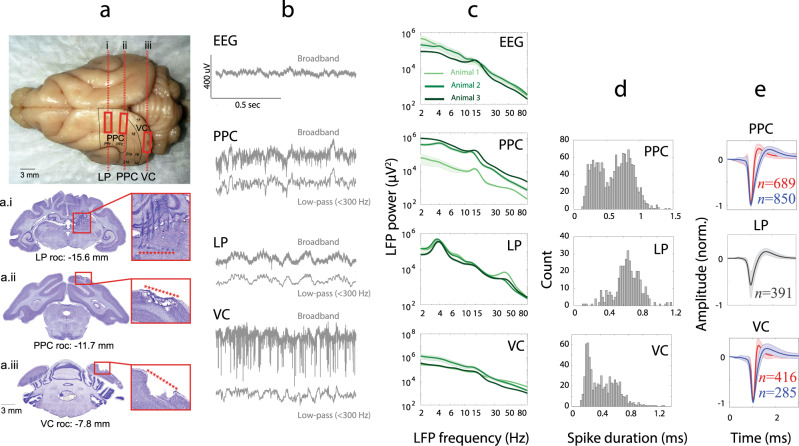
Histology and endogenous alpha-band oscillations in awake head-fixed ferrets. Histology verification of implant locations. **a** Ferret brain with marks (highlighted by rectangles) left by microelectrode array implants in LP, PPC, and VC regions. Outlines of cortical fields are based on a published ferret neuroanatomical atlas^67^. Dashed lines indicate the location of cell-stained coronal cross-sections through the center of the multi-electrode array implanted sites shown below (i–iii). Corresponding anterior–posterior atlas coordinates are indicated below each section in mm, relative to the occipital crest. a.i Coronal cross-section through the LP implant region shows implant electrode tracks and endpoints (dark spots of cell agglomerations above red stars). a.ii Coronal cross-section through the PPC implant region demonstrates the tissue damage at the site of the electrode array caused by the removal of the implant during brain extraction (below red stars). a.iii Coronal cross-section through the VC implant region marked by tissue loss (below red stars) caused by the removal of the implant during brain extraction after completion of the experiment. “roc” indicates “reference to occipital crest”. **b** Sample 1-s length EEG, broadband extracellular recordings and local field potential signals (LFPs) from the posterior parietal cortex (PPC), visual cortex (VC), and lateral posterior nucleus/Pulvinar complex (LP) from a head-fixed awake ferret (resting state, no task performed by animal). **c** Spectral analysis of EEG and LFPs showing the spectrum (mean ± SEM) with error bars in the light background from three animals (each animal in a different shade of green). The scalp EEG signal demonstrated a weak alpha-band peak for all animals. PPC showed clear alpha-band activity (12–16 Hz) for all animals. LP showed prominent theta, gamma (35–50 Hz), and a weak alpha activity for all animals. A weak alpha-band activity is also evident for the visual cortex (VC) for all three animals. A closer look at PPC spectra indicates slight differences in the individual alpha frequency between animals (14.42, 12.32, 14.42 Hz for animals 1, 2, and 3 respectively). **d** Histogram of spike duration was defined as the time from trough to peak in PPC, LP, and VC. **e** Color-coded spike waveforms (mean ± SEM) were calculated for two identified clusters of narrow-spiking (red, *n* = 689 in PPC, *n* = 416 in VC) and broad-spiking (blue, *n* = 850 in PPC, *n* = 285 in VC) neurons in PPC and VC. LP neurons comprised only one cluster (*n* = 391).

We hypothesized that the effect of tACS on network dynamics was a result of the synergistic interaction of endogenous network activity and the exogenous electric field delivered by tACS. Thus, understanding the endogenous network dynamics was a necessary step to identify the stimulation targets before examining their engagement by tACS. We first characterized the endogenous oscillations to validate the existence of previously reported alpha oscillations in the thalamo-cortical system of awake head-fixed ferrets^42^. The extracellular broadband signals were recorded from implanted microelectrode arrays in PPC, VC, and LP regions (Fig. 1a). The post-mortem histological investigation verified the correct implant locations (Fig. 1 a.i–iii).

The local field potential (LFP) signals were acquired by low-pass filtering (<300 Hz) the extracellular broadband recordings (Fig. 1b). Spectral analysis of cranial screw EEG and LFP signals in PPC, VC, and LP showed an alpha peak (12–16 Hz) in PPC, VC, LP and EEG recordings for all three animals (Fig. 1c). An alpha oscillation was defined as an increase in power (a “bump”) beyond the 1/*f* power distribution that permeates the background of electrophysiological recordings^43^. The alpha peaks were strongest in PPC and VC, with slight differences in individual alpha peak frequencies between animals: 14.42, 12.32, 14.42 Hz for animals 1, 2, and 3, respectively. Prominent theta oscillations (3–5 Hz) were evident in LP for all animals. To analyze the endogenous oscillations at the neuronal level, we extracted the extracellular spikes (*n* = 1539, 701, 391 units in PPC, VC, and LP, respectively), and clustered the unit waveforms based on spike duration (spkD) defined as trough-to-peak time. We identified two populations of neurons in PPC (narrow spiking, *n* = 689: spkD = 0.361 ± 0.005 ms, broad spiking, *n* = 850: spkD = 0.810 ± 0.005 ms) and VC (narrow spiking, *n* = 416: spkD = 0.283 ± 0.005 ms, broad spiking, *n* = 285: spkD = 0.651 ± 0.009 ms), but only broad spiking neurons in LP (*n* = 391, spkD = 0.642 ± 0.008) (Fig. 1b, c). We next examined how different neurons from each region interacted with each other and with the meso- and macroscale neuronal signals (LFP and EEG, respectively).

### Population coupling is stronger for narrow-spiking neurons and oscillates at alpha frequency

**Fig. 2:**
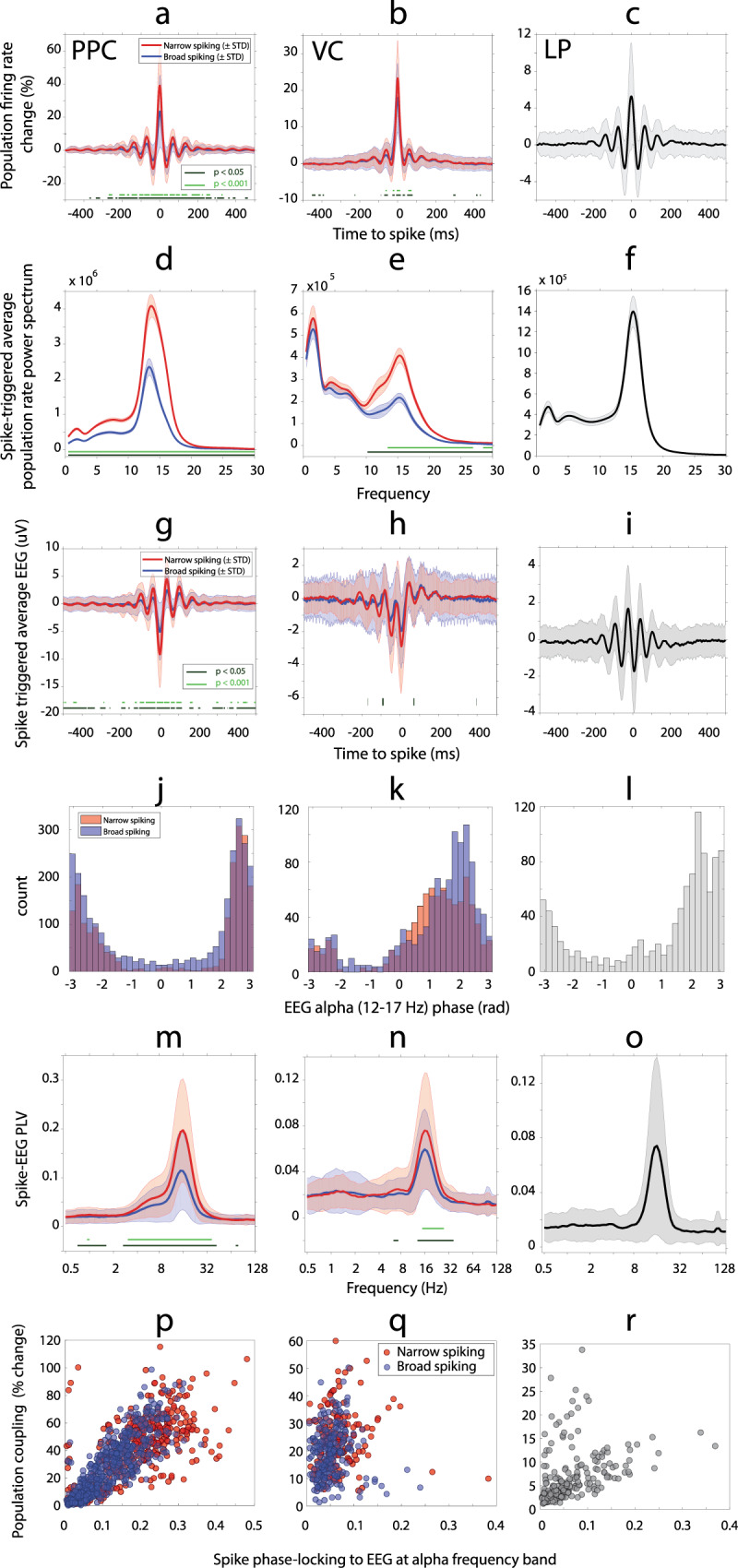
Population coupling and spike-EEG coupling are stronger for narrow-spiking neurons and oscillate in alpha-frequency band. **a**–**c** Percentage-change of spike-triggered population firing rate (mean ± STD) as a function of time-to-spike. The percentage-change is computed relative to the corresponding baseline value at *t* = −500 ms in PPC, VC, and LP. The spike-triggered population firing rate functions have larger amplitude at spike instant (time lag zero) for narrow-spiking units (red) compared to broad-spiking units (blue) in both cortical regions (two-sample *t*-test between broad and narrow-spiking units at each time point at two significant levels indicated by horizontal green lines). **d**–**f** The power spectrum (mean ± SEM) of the spike-triggered population firing rate in PPC, VC, and LP. A prominent peak in the alpha (11–17 Hz) frequency band is evident for all three regions. The alpha power is higher for narrow-spiking units (red) when compared to broad-spiking units (blue) in both cortical regions (two-sample *t*-test at each frequency point at two significant levels indicated by horizontal green lines). **g**–**o** Alpha synchronization between EEG and spikes, and prominent engagement of narrow-spiking neurons. **g**–**i** Spike-triggered EEG (mean ± STD) as a function of the time-to-spike in PPC, VC, and LP. Spike-triggered average EEG shows an oscillatory pattern for all three regions and shows larger amplitudes at peaks and troughs for narrow-spiking (red) when compared to broad-spiking (blue) neurons (two-sample *t*-test at each time point at two significant levels indicated by horizontal green lines). **j**–**l** The histogram of phase preference of spiking units in PPC, VC, and LP as a function of the instantaneous phase of the alpha-band (12–17 Hz) EEG oscillation. Color denotes the counts for narrow- and broad-spiking neurons. Two classes of neurons show similar phase preference in PPC (Broad-spiking neurons: 3.02 ± 0.02 rad, Narrow-spiking neurons: 3.05 ± 0.02 rad), and slightly different phase preference in VC (broad-spiking neurons: 1.91 ± 0.03 rad, narrow-spiking neurons: 1.64 ± 0.04 rad). **m**–**o** Phase-locking between single units and EEG measured by PLV (mean ± SEM) as a function of EEG frequency. Synchronization was increased across a broad range from 3 to 32 Hz range with a prominent peak in the alpha (12–17 Hz) frequency range for recorded cortical and thalamic sites. The narrow-spiking cells (red) show significantly higher PLV compared to broad-spiking units (blue) (two-sample *t*-test at each frequency point with *p*-values < 0.05 indicated by horizontal line). **p**–**r** Population coupling of single neurons and their engagement with globally measured alpha correlate strongly in PPC but not in VC and LP. Significant correlations were tested by a two-sided Pearson correlation test. No multiple comparison adjustment for *p*-value was performed. **p** Population coupling of single units versus their engagement by alpha-band EEG is plotted for PPC. There is a strong correlation between population coupling and large-scale alpha synchronization for narrow-spiking (red, *n* = 403, Pearson correlation test, *r* = 0.71, 2-sided *p* = 1.3*e^−61^, 95% CI [0.66, 0.75]) and broad-spiking units (blue, *n* = 582, Pearson correlation test, *r* = 0.86, 2-sided *p* = 3.46*e^−168^, 95% CI [0.84, 0.88]) in PPC. Color denotes the neuron type. **q** Population coupling of single units versus their engagement by alpha-band EEG is plotted for VC. Population correlation and large-scale alpha synchrony do not correlate in VC (narrow-spiking: red, *n* = 173, Pearson correlation test, *r* = 0.06, 2-sided *p* = 0.46, 95% CI [−0.09, 0.21], and broad-spiking: blue, *n* = 221, Pearson correlation test, *r* = 0.01, *p* = 0.88, 95% CI [−0.12, 0.14]). **r** Population coupling of single units versus their engagement by alpha-band EEG is plotted for LP. This analysis revealed that population coupling and long-range synchrony are weakly correlated in LP (*n* = 251, Pearson correlation test, *r* = 0.35, 2-sided p = 3.5*e^−7^, 95% CI [0.22, 0.46]). The values on the vertical axis for all figures are the percentage-change in spike-triggered population firing rate at the time of spike generation relative to the corresponding baseline value at *t* = −500 ms.

We recorded broadband extracellular activity from cortical and thalamic regions and the EEG signal to measure endogenous oscillations. This recording enabled us to examine the interaction between neural activity at the level of single units and network activity at the level of the LFP or EEG to better understand the underlying mechanisms of the endogenous oscillations. We first asked if there was a relationship between single-unit activity and the local dynamics in each region by measuring population coupling^44^. We constructed the population firing rate function for each region of interest (ROI) based on its spiking activity. Spike-triggered average population firing rates were extracted at the occurrence of individual spikes for all unit types by region and given as a percentage of the baseline value in the 500 ms before spike generation. The within-region average of this value provided a measure of how strongly each unit was coupled to local oscillations (Fig. 2a–c)^44^. We observed that the coupling of single units to the population rate in all regions oscillated in alpha-frequency range, confirmed by spectral analysis. We found a prominent peak in the alpha (11–17 Hz) frequency band for all three regions (Fig. 2d–f). A weaker theta-band (3–8 Hz) oscillation was also evident in all regions [defined as an increase above background noise]. Comparing the narrow- and broad-spiking neurons, we found that narrow-spiking neurons had stronger population coupling than broad-spiking neurons (Fig. 2d–f, amplitudes at time lag zero), and the fluctuation of the coupling had more oscillatory power over a wide frequency range including the alpha band in both PPC and VC (Fig. 2d–f).

### Alpha synchronization between EEG and spikes, and prominent engagement of narrow-spiking neurons

Next, we examined alpha synchrony at the network scale by quantifying the synchronization between single units in all three ROIs and EEG oscillations. The average spike-triggered EEG showed an oscillatory pattern at alpha-frequency band (Fig. 2g–i). Further analysis showed a non-uniform phase histogram for each region (Fig. 2j–l). Narrow-spiking PPC units preferred to spike at 174.67°, confidence interval (CI) = [172.71°, 176.63°] phase of EEG alpha oscillations. The preferred phase for broad-spiking neurons was 173.14°, CI = [170.95°, 175.32°]. For VC neurons, the preferred phase was slightly different between narrow (94.06°, CI = [89.50°, 98.62°]) and broad spiking (109.63°, CI = [105.48°, 113.77°]) neurons. The preferred synchronization phase for LP units was 142.63°, CI = [138.44°, 146.82°]. We extracted the phase-locking value (PLV) to examine the strength of spike-to-EEG phase-locking as a function of frequency. While the PPC units were phase-locked to EEG at a wide frequency range of ~3–32 Hz (Fig. 2m), the peak in PLV was limited to a narrower band of ~10–32 Hz for VC (Fig. 2n) and LP units (Fig. 2o). Synchronization in the alpha-frequency band (12–17 Hz) was evident for single units of all three ROIs. We found that the phase-locking between EEG and single units was stronger for narrow-spiking neurons when compared with broad-spiking ones. This pattern was evident in both PPC and VC (Fig. 2m, n).

We further analyzed the relationship between population coupling of single neurons (as shown in Fig. 2a–f) and spike phase-locking to more global EEG alpha oscillations (Fig. 2g–o). We found a strong correlation between population coupling and spike phase-locking to EEG signal in alpha-frequency band in PPC, no correlation in VC, and weak correlation in LP (Fig. 2p–r). In summary, these results indicate that the more a PPC unit was coupled to the population dynamics, the more it was coupled to the macroscopic alpha oscillation measured by EEG. Conversely, the less a PPC unit was coupled to local dynamics, the less it was coupled to the alpha oscillations measured by EEG.

### Synchronization and directed functional connectivity in thalamo-cortical network

**Fig. 3:**
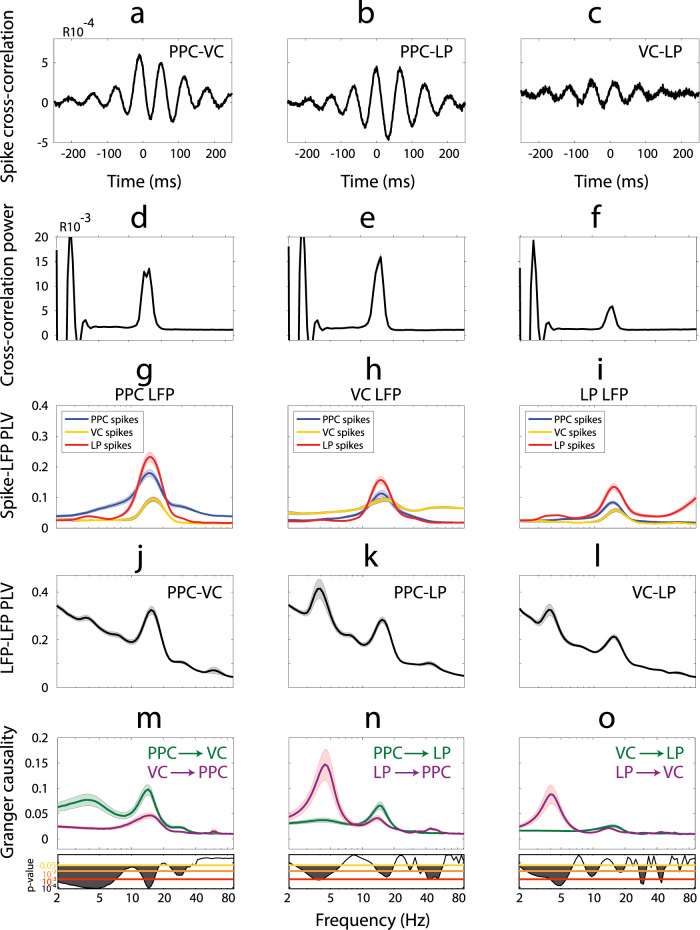
Synchronization between brain regions and directed functional connectivity in thalamo-cortical network. Population-averaged spike cross-correlation measured between **a** PPC and VC, **b** PPC and LP, and **c** VC and LP. The mean power spectrum of spike-correlations displayed a prominent peak in the alpha band (centered on ~15.23 Hz) for PPC–VC (**d**), PPC–LP (**e**), and VC–LP (**f**) spike cross-correlations. **g** Phase-locking between spikes from PPC, VC, and LP and the LFP from the PPC region. **h** Phase-locking between spikes from PPC, VC, and LP and the LFP from VC. **i** Phase-locking between spikes from PPC, VC, and LP and the LFP from LP. **j** Phase-locking value (PLV) between the LFP recorded from PPC and VC regions as a function of frequency indicates synchronization in the alpha-frequency band. **k** PPC and LP are synchronized in both the theta and alpha band. **l** Theta and alpha-band synchronization are evident between VC and LP regions. **m** Spectrally resolved Granger causality shows that the influence of PPC on VC (green) is larger than the influence of VC on PPC (violet) in all examined frequencies including the alpha band (2-sided *t*-test, indicated by the exact *p*-values below the *p* = 0.05 line (yellow)). **n** LP has a causal influence on PPC at theta frequency range (violet). In return, PPC has a causal influence on LP at the alpha-frequency band (green). **o** LP drives VC at theta frequency range (violet). VC and LP drive each other comparably (2-sided *t*-test, indicated by the exact *p*-value around the *p* = 0.05 line (yellow)) at alpha-frequency range. **g**–**o** Each recording session was treated as an independent sample unit. All measures are shown as mean (thick line) ± SEM (light background).

To better understand the dynamics of endogenous network oscillations, we examined the synchronization between all three region pairs using spiking and LFP activity. We first looked at the synchronization of spiking activity between recording regions. The population-averaged spike cross-correlations demonstrated synchronous oscillatory structure occurring within the alpha-frequency band between all three region pairs (Fig. 3a–c). The mean power spectrum (±SEM) of cross-correlations indicated a prominent peak in the alpha band (centered on ~15.23 Hz, Fig. 3d–f). These results showed that spiking activity in PPC, VC, and LP was synchronized in the alpha frequency. We next examined the phase-locking between spikes and the LFP for each region pair to understand the between-region synchronization. The spikes from all three ROIs were significantly phase-locked to the alpha oscillation in the LFP from PPC (Fig. 3g), VC (Fig. 3h), and LP (Fig. 3i). We computed population average phase-locking between LFPs obtained from three regions to understand their interactions (see Supplementary Fig. S1 for individual animals). We found strong phase-locking in the alpha range (peak frequency at 15.23 Hz) and weak synchronization in the theta range (peak value at 3.75 Hz) between the two cortical regions (Fig. 3j). When considering the synchronization between one of the two cortical regions and LP, we found strong phase-locking at both theta and alpha frequencies (Fig. 3k, l). The theta and alpha synchronization between PPC and LP was larger when compared to the corresponding values between VC and LP. These results indicate that the two cortical regions synchronize in the alpha range, while the synchronization between the cortical regions and LP occurred in both alpha and theta frequency ranges as previously reported^42^. Further analysis by population average conditional Granger causality measure (cGC) showed that alpha synchronization between cortical regions was more directed from PPC to VC than the opposing direction (Fig. 3m, see Supplementary Fig. S1 for cGC measures for individual animals). The results of directed functional connectivity analysis also suggested that LP influenced both cortical regions in the theta range, and to a smaller extent that the PPC drove the LP in the alpha range (Fig. 3n, o). Our results did not indicate a preference in the directionality of alpha oscillations between VC and LP (Fig. 3o). Taken together, these results showed that alpha oscillations originated primarily in PPC and propagated to both VC and LP, whereas theta oscillations originated primarily in LP and propagated to the other two regions.

### Measurements and modeling of tACS electric field in the ferret brain

We identified endogenous alpha oscillations that originated in PPC as a potential target for transcranial stimulation. To examine the effect of tACS on neurons, we approximately matched the electrode current density of human tACS. The tACS current density is ~1 µA/mm^2^ per electrode for a typical dual-site human tACS protocol in which the current is 2 mA zero-to-peak^21^. We trimmed carbon-silicone tACS electrodes used in human studies to make smaller circular electrodes with a radius of $r=5{\rm{mm}}$ to apply to the ferret head. To achieve a current density comparable to values used in human settings, we used current amplitudes up to 80 µA zero-to-peak in our experiments.

**Fig. 4:**
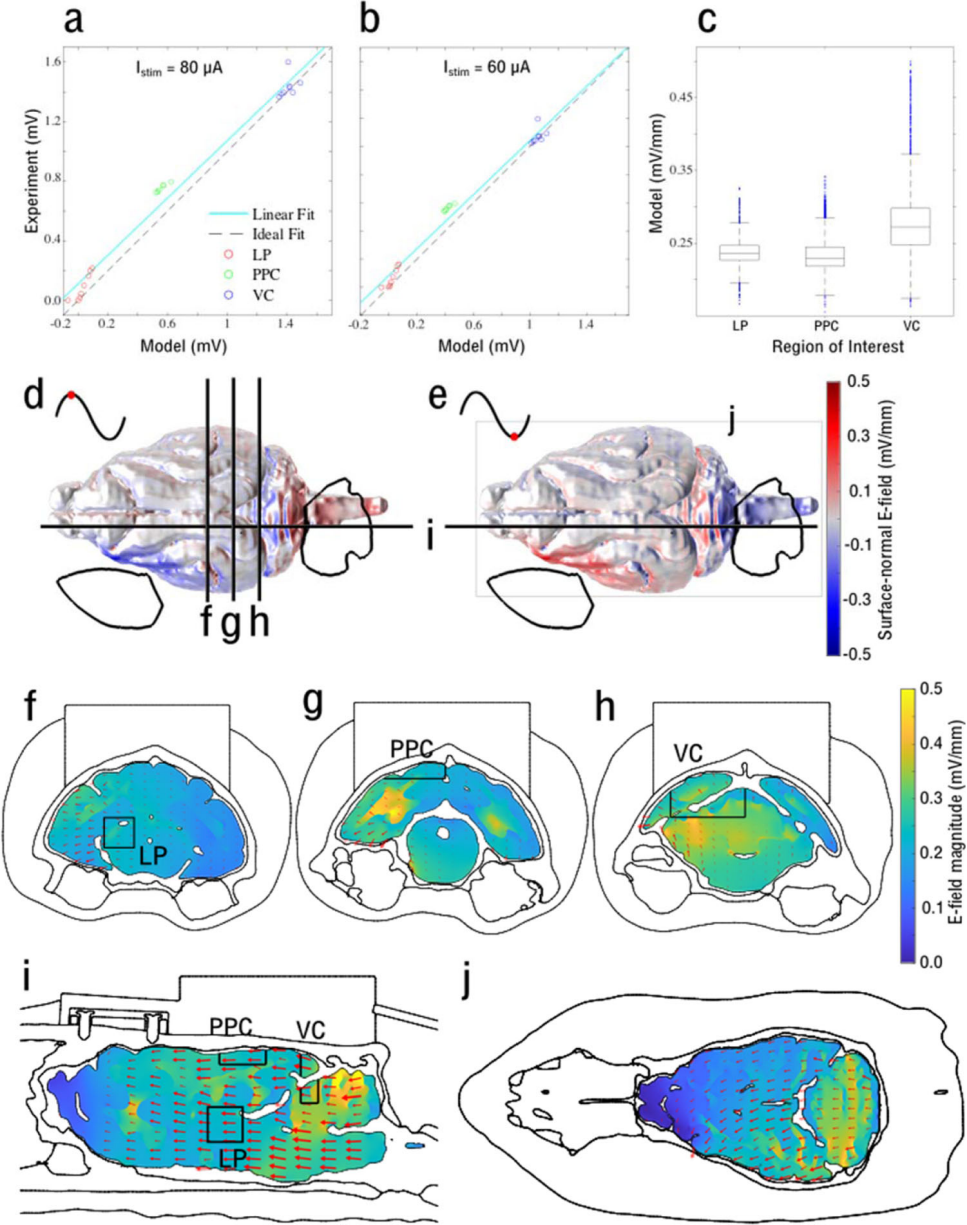
Measurement and modeling of the tACS electric field in the ferret brain. **a**, **b** Comparison between recorded and simulated electric potentials using stimulation current intensity of 80 µA and 60 µA in three recording sites: LP, PPC, and VC with 8, 6, and 8 grid electrodes, respectively. The *y* axis represents the experimental values averaged over *n* = 6 measurements (2 blocks and 3 electrode reference schemes), while the *x* axis shows the corresponding potential value from the model. The solid cyan line represents the linear regression of the data, which ideally should match the dashed black unity line. **c** Distribution of the electric field magnitude in the three ROIs, predicted by the model. The central mark in each box indicates the median, and the bottom and top edges indicate the 25th and 75th percentiles, respectively. The whiskers extend to the most extreme data points not considered outliers (99.3% of the data), and the outliers are plotted as blue dots. The number of samples in each ROI correspond to the number of finite elements: *n* = 2327, 8782, and 7944 for LP, PPC, and VC, respectively. These data demonstrate a relatively weak electric field of ~0.25 mV/mm in the ROIs. Source data are provided as a Source Data file. **d**, **e** Spatial distribution of the magnitude of the electric field surface-normal component (min: −0.497 mV/mm, max: 0.507 mV/mm) on the brain surface at, respectively, 90° and 270° phase of the tACS wave (red dots on a cycle of sinewave). The electric field magnitude is greatest in areas proximal to stimulation electrodes (open black circles in left frontal and midline occipital regions). The direction of the surface-normal electric field flips according to the tACS phase. **f**–**j** The electric field distribution along three coronal sections (including ROIs), one sagittal section (horizontal lines in **d** and **e**), and a transversal section is displayed in **f**–**j**. The model prediction shows that the electric field magnitude is <0.5 mV/mm in all sections. The maximum electric field (bright yellow) occurs outside the ROIs. Arrows representing the direction of the electric field components are scaled in length by the electric field strength in the plane of the sections in **f**–**j** and demonstrate dominant posterior–anterior electric field direction.

We examined what electric field strengths were generated inside the ferret brain when applying tACS currents with amplitudes ≤ 80 µA. To estimate the electric field magnitude in the ROIs and the entire ferret brain, we developed a computational model of the current flow in the ferret head (Supplementary Fig. S5). The model included detailed anatomical structures, head-post, bone screws, dental cement, and surgical craniotomies based on MRI and CT scan data and tissue conductivities from the literature (Supplementary Table S1). We validated the model adequacy by comparing its predictions to experimentally measured electric potentials for stimulation currents of 80 and 60 µA. The electric potentials were measured with the same implanted electrode arrays used for the electrophysiology recordings. The model prediction for the electric potentials was highly correlated with the experimental data (Fig. 4a, b respectively, correlation coefficient *r* = 0.99, *p* < 0.0001 for both validations). The error bars of each measurement in the plots stem from variation across the six sets of recordings corresponding to two recording blocks and three different reference electrode schemes, after re-referencing the data and excluding electrodes with poor signal quality (see “Methods”/“Electric potential measurements” section for details). The simulated distribution of the electric field magnitude in the three ROIs is summarized in Fig. 4c. The estimated electric field magnitude was slightly higher for the VC region compared to PPC and LP, but most of the magnitudes across all ROIs fell into a relatively narrow range of 0.22–0.30 mV/mm. Figure 4d, e shows the surface-normal component of the electric field on the brain surface (see also Supplementary Fig. S6 for more details), corresponding to peaks and troughs of the tACS current cycle. The areas underneath the stimulation electrodes (indicated by large open circles on the left frontal and medial occipital regions, see also Supplementary Fig. S8) showed maximum field strength with directions flipped according to the stimulation wave polarity. Figure 4f–i visualizes the electric field strength and direction in deeper cortical and sub-cortical regions (for current density see also Supplementary Fig. S7) across coronal and sagittal sections including the implanted recording sites. As expected, the direction of the electric field was predominantly along the posterior–anterior axis as shown by red arrows in the coronal and sagittal sections.

These results indicated that neither the surface-normal nor the total magnitude of the tACS electric field exceeded 0.53 mV/mm in the brain. The modeling results also indicated that the arrangement of the tACS electrodes in our experiments did not necessarily result in maximum electric field in the PPC, VC, and LP regions where all cortical recordings were obtained. The electric field strength was in the range of values reported for humans and non-human primates in models and experiments^9,45^. These results supported our decision to use 80 µA as stimulation intensity to produce comparable electric fields at the site of the implanted electrodes as in human tACS experiments.

### Transcranial alternating current stimulation engages cortical alpha oscillations at the single neuron level

We found that synchronization between spikes and both local (Fig. 2a–f) and network-scale oscillations (Fig. 2g–o) was highest for alpha-band oscillations across all three animals. In addition, we identified the PPC as the source of alpha oscillations that drives VC and LP (Fig. 3). These results suggested that alpha oscillations were the ideal candidate target for tACS. Furthermore, we identified that tACS with an amplitude of 80 µA resulted in a weak electric field of <0.5 mV/mm across the entire ferret brain that was comparable to tACS in humans. Thus, we performed a comprehensive series of experiments in which we applied tACS with different frequencies and amplitudes and simultaneously measured the spiking activity in PPC, VC, and LP regions. Nine stimulation frequencies centered on each animal’s endogenous alpha frequency (EAF) (${f}_{{\rm{stim}}}=\left[-4,-3,-2,-1,0,1,2,3,4\right]+{EAF}$) and 6 amplitudes $({I}_{{\rm{stim}}}=\left[5,10,15,20,40,80\right]\mu {\rm{A}},{\rm{peak}}\; {\rm{values}})$ were applied over multiple sessions per animal (*n* = 16, 21, 14 sessions for animals 1–3, respectively). We hypothesized that if entrainment was the underlying mechanism of how tACS affects neural activity, then the synchronization between endogenous oscillations and external tACS waveform should increase as the tACS frequency approaches the endogenous alpha frequency. This increased synchrony between an external periodic perturbation and an oscillator is a well-known phenomenon in dynamical systems; as the amplitude of the stimulation is increased, the range of frequencies for which entrainment occurs is also increased. This relationship results in a triangular Arnold tongue region with an increased coupling on a two-dimensional heat-map that plots the amplitude (*y* axis) and frequency (*x* axis) of stimulation^34^.

**Fig. 5:**
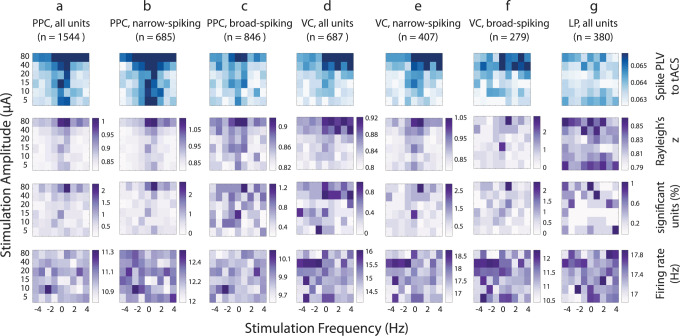
Transcranial alternating current stimulation engages cortical alpha oscillations at the level of individual neurons. Synchronization maps (blue) and corresponding Rayleigh’s *z*-score (purple, second row), percentage of units with significant phase-locking value (purple, third row), and firing rate maps (purple, bottom row) as a function of stimulation parameters for PPC (**a**–**c**), VC (**d**–**f**), and LP (**g**). The synchronization maps show phase-locking between individual spikes and tACS wave as measured by phase-locking value (PLV) averaged across units. The horizontal axis indicates the distance (in Hz) from individual alpha frequency, and the vertical axis shows the stimulation amplitude. **a** (top two rows) tACS entrains PPC units as indicated by the Arnold tongue centered at the individual endogenous alpha frequency. **b**, **c** (top two rows) Narrow-spiking units in PPC show stronger phase-locking to tACS when compared to their broad-spiking counterparts. **d**–**f** (top two rows) VC units are phase-locked to tACS, but the area of entrainment is asymmetric and favors stimulation at frequencies above the endogenous peak frequency. **g** (top two rows) The map for LP units does not show prominent synchronization. **a**–**g** (third row) Only a small percent of units (<2%) show significant modulation of spike phases as measured by Rayleigh’s test. **a**–**g** (bottom row) The random pattern of firing rate maps for all regions/unit types indicates that tACS did not modulate the firing rate of target neurons.

First, we analyzed the phase-locking of all spikes to the tACS waveform regardless of spike shape in a pooled data set for all three animals (see Supplementary Figs. S2–S4 for individual animals). A triangular region centered on the endogenous alpha frequency was found for PPC units (Fig. 5a top, synchronization maps, darker shades of blue indicate higher PLV). We evaluated the modulation of spike phases by tACS using Rayleigh’s test for non-uniformity of circular data and found that the *z*-statistics also displays a triangular shape for PPC neurons (Fig. 5a, second row). The percentage of PPC units with a significant phase modulation was low (<2%, Fig. 5a, third row). Furthermore, the firing rates of PPC neurons displayed a random modulation as a function of stimulation waveform and did not display the triangular, Arnold tongue, shape. These analyses confirmed that the applied tACS amplitude was subthreshold and did not modify the firing rate of the units (Fig. 5a bottom). To differentiate the cell-type-specific effect of tACS, we extracted the synchronization maps (i.e., spike PLV to tACS as a function of tACS frequency and amplitude) for narrow- and broad-spiking neurons separately. We performed the same analysis on clustered units in PPC based on spike shapes: the narrow-spiking neurons (*n* = 685) displayed darker triangular regions when compared to their broad-spiking counterparts (*n* = 864) (Fig. 5b, c, top). The synchronization map between VC units and tACS (for collective or clustered spikes) did not show a triangular Arnold tongue shape (Fig. 5d–f top) as observed for PPC units. Instead, a weak modulation was observed for frequencies close to the endogenous alpha frequency, and became more evident for stimulation amplitudes above 20 µA and was skewed toward frequencies higher than endogenous alpha as indicated by the dark region on the top right corner in Fig. 5d, e. We did not find synchrony regions for LP neurons (Fig. 5g, top). As for PPC, Rayleigh’s *z*-statistics in VC and LP (Fig. 5d–g, second row) showed a similar pattern to corresponding PLV maps (Fig. 5d–g, top), and the percentage of units showing significant phase modulation was low (<2%, Fig. 5d–g, third row). Similar to PPC, tACS did not modulate the firing rate of individual units in VC or LP (Fig. 5d–g, bottom row). Taken together these results show the synchronization map between single units and tACS that induced an electric field of < 0.5 mV/mm across the ferret brain. The synchronization region follows a triangular Arnold tongue shape, with phase-locking values that are just slightly higher than their neighbor regions. The PPC units exhibited an Arnold tongue pattern indicative of their weak entrainment by tACS. The VC units demonstrated a partial Arnold tongue, and no regular pattern of synchrony was observed for LP neurons. Among narrow- and broad-spiking neurons, the former ones showed more defined Arnold tongue regions.

### Peripheral nerve alternating current stimulation fails to entrain PPC neurons in a frequency- and amplitude-dependent way

Since tACS was applied to the scalp, it was important to consider whether the entrainment of neurons was a direct effect from the electric field delivered to the brain or an indirect effect from the activation of peripheral nerves. To control for peripheral nerve activation, we applied the identical tACS paradigm to the shaved lower back of two ferrets (Supplementary Fig. S8a). If the Arnold tongue patterns in Fig. 5 were a result of peripheral nerve activation, we would expect to see a similar Arnold tongue pattern in this experiment where the skin, but not the brain, was stimulated. We used the identical analysis pipeline and again extracted two types of neurons based on the waveform of the action potentials (Supplementary Fig. S8b) similar to the findings in the main experiment (Fig. 1e, PPC). However, in stark contrast, we did not find a triangular region centered on the endogenous alpha frequency indicative of the Arnold tongue for PPC units. Instead, we observed a more random pattern of PLV (Supplementary Fig. S8c–e, top row), Raleigh’s z distance (Supplementary Fig. S8c–e, second row), percent of significant units (Supplementary Fig. S8c–e, third row), and firing rate (c–e, bottom row), in both unit types (d, e). This suggests that peripheral nerve stimulation is not the underlying mechanism of action of tACS.

### Repeated tACS sessions do not produce a long-lasting effect on neuronal synchrony

Due to the long session duration, we investigated whether there is any long-lasting effect on neural synchrony after repeated tACS sessions. To study this, we conducted a separate experiment of 14 repeated tACS sessions (*n* = 1 ferret). Both spiking and LFP data were recording from PPC, VC, and LP using multi-electrode arrays. The tACS electrode locations (Supplementary Fig. S9a) were identical to the main tACS experiment that showed the Arnold tongue. To rigorously test the potential confound of cumulative changes to the spiking behavior due to repeated stimulation for determining the Arnold tongue, we stimulated at a single frequency (endogenous peak alpha frequency, 14.5 Hz) and at the highest amplitude investigated in this study (80 µA), which presumably would maximize the long-lasting effect if there were any. We recorded resting-state data before the onset of tACS, followed by 20 or 40 min of continuous tACS, and then recorded post-stimulation resting-state. Of note, the 20 or 40 min of stimulation was much longer than the 90 s per condition in the tACS paradigm that showed the Arnold tongue. Thus, if no long-lasting effect were found for this paradigm, then it could be assumed that carry-over effects did not significantly affect the emergence of the Arnold tongue. Phase-locking values (PLV) between spiking in single units and instantaneous alpha-frequency phase in LFP were calculated for the resting-state periods before and after stimulation. We did not see a significant increase in PLV compared to baseline, which indicated that there was no long-lasting effect of the stimulation session in terms of promoting neuronal synchrony (Supplementary Fig. S9b). Therefore, the Arnold tongue that we found was not the result of a cumulative effect of stimulation, but rather a reflection of the instantaneous effects of stimulation. The cause for persistent effects of tACS on neural activity that are reported in the human literature thus remains an open question.

### Local rhythmic stimulation in PPC engages cortical alpha oscillations at the level of individual neurons

Due to the potential lack of spatial specificity of tACS, we cannot rule out the possibility that entrainment of neural activity in PPC was an indirect effect due to modulation of activity in another region. To further clarify the potential direct and indirect effect of tACS on neural activity and whether broad network-level stimulation was required to drive neural synchrony, we conducted two control experiments to localize the stimulation in PPC, in contrast to the spatially distributed electric field induced by scalp tACS. In the first experiment, we applied ACS through bones screws (instead of tACS to the scalp) that flanked the PPC in the anterior–posterior direction (Supplementary Fig. S10a) to maximize stimulation of PPC while sparing VC (*n* = 3 animals). In the second experiment, we applied AC-modulated optogenetic stimulation of PPC (Supplementary Fig. S11a, *n* = 2 animals). Given the high spatial specificity of optogenetic stimulation by virtue of the localized viral injection, this experiment provided additional insights on the role of local stimulation.

In the bone-screw stimulation experiment, the stimulation site, i.e., PPC, showed increased spike-field PLV as stimulation amplitude increased. It should be noted that the PLV values for PPC were much higher when compared to tACS as a result of the more localized stimulation and the higher current density due to the small surface area of the bone screws compared to the tACS electrodes. In contrast to the scalp tACS experiments, this more localized stimulation caused broad synchronization of neural activity to the stimulation waveform across different frequencies, which may be an indication of a ceiling effect. Such an observation is predicted by the Arnold tongue, where higher stimulation amplitudes do not show a dependence of the synchronization on the stimulation frequency (i.e., top of an Arnold tongue, where the synchronization is strong across all stimulation frequency, Supplementary Fig. S10d).

Importantly, the investigation of neural activity in VC provides additional insights since VC received stimulation in the tACS but not the bone-screw experiments. In particular, this additional experiment provides a unique opportunity to dissociate the direct tACS effect from a more indirect effect through network interactions. The neural synchronization in VC showed a preference for high amplitude and high-frequency bone-screw stimulation that was higher than the endogenous alpha frequency (Supplementary Fig. S10c). This pattern resembled the result of tACS in VC under high amplitude and frequency above the endogenous peak (Fig. 5d, top two rows). This suggests that this preference might be due to a network-related phenomenon induced by PPC–VC connectivity. On the other hand, since only the tACS (but not bone-screw) stimulation-induced stronger spike-field synchrony around the endogenous alpha frequency in VC, it implies that this effect does require direct stimulation. In another word, the Arnold tongue-like pattern was not evoked by network stimulation (through the PPC–VC connection).

In the optogenetic stimulation experiment, we modulated the amplitude of the laser input in a sinusoidal waveform of different amplitudes and frequencies (Supplementary Fig. S11a) to mimic the setup of the tACS experiment. We implemented the same stimulation paradigm as in the tACS experiment (randomly interleaved frequency and amplitude combinations). We found that PPC units exhibited a triangular Arnold tongue pattern in both the synchronization map and spike-phase modulation map (Supplementary Fig. S11b). This pattern was similar to the scalp tACS experiment (Fig. 5a), indicating a common underlying principle for entrainment by the AC-modulated optogenetic stimulation and tACS. The PLV value here (from 0.128 to 0.14, Supplementary Fig. S11b, top) was higher than the tACS weak entrainment (from 0.063 to 0.066, Fig. 5, top row), which is not surprising given that the biophysical mechanisms for optogenetic (directly opens ion channels) and electric stimulation (polarizes neuron by an electric field) are different. However, despite these inherent differences, we have replicated the general Arnold tongue shape of the synchronization map. Taken together, these results show that tACS was able to entrain single units in an Arnold tongue pattern that was similar to the one induced by sinewave modulated optogenetic stimulation. Therefore, we propose that the Arnold tongue is indeed a general principle of how oscillating brain networks respond to weak periodic perturbations.

### Endogenous alpha oscillations in a biophysical model of thalamo-cortical network and dynamics of synchronization by tACS

**Fig. 6:**
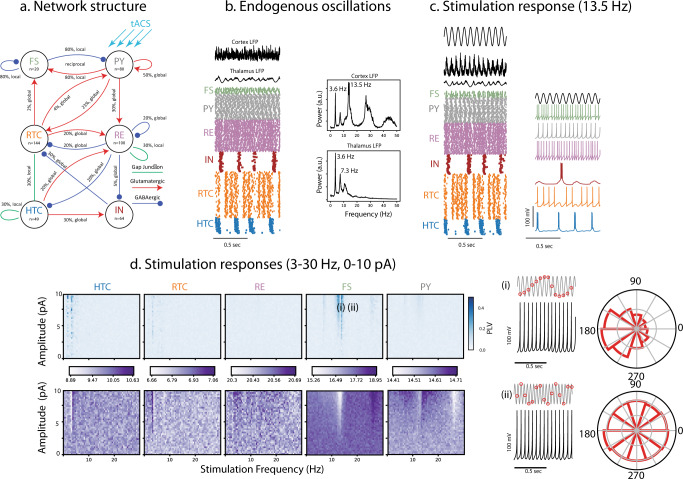
Endogenous alpha oscillations in a thalamo-cortical model and synchronization by tACS. **a** The structure of the thalamo-cortical network with excitatory, inhibitory, and gap-junction connections between neuronal populations. The cortical network includes fast-spiking inhibitory (FS) and regular-spiking pyramidal (PY) neurons. The thalamic network includes relay thalamo-cortical (RTC), reticular (RE), high-threshold bursting (HTC), and thalamic local inhibitory (IN) neurons. The tACS is applied only to pyramidal neurons (blue arrows). **b** Endogenous oscillations shown by (top) 1-s cortical and thalamic local field potential (LFP) traces, and (bottom left) the raster plot with each dot representing the firing instant of a neuron. (Bottom right) The cortical LFP has a dominant spectral peak at the alpha range (13.5 Hz), and a smaller peak at theta range (3.6 Hz). The thalamic LFP has a dominant theta peak (3.6 Hz), its harmonic (7.3 Hz), and a weak alpha peak (13.5 Hz). **c** Network response to a 13.5 Hz, 8 pA tACS. The cortical and thalamic LFP traces, and the raster plot showing the firing of all neurons (left), and the membrane voltage traces of sample neuron from each cell type (right). **d** Network response to a group of stimuli with frequencies between 3 and 30 Hz, and amplitudes between 0 and 10 pA. Top left heat-maps: The color-coded phase-locking value (PLV) as a function of stimulation frequency (horizontal axis), and stimulation amplitude (vertical axis) for each neuron type. Darker colors indicate higher synchronization between individual neurons and tACS with corresponding frequency and amplitude. The FS and PY neurons show triangular-shape high PLV regions centered on the alpha peak frequency (13.5 Hz). Bottom left heat-maps: The firing rate (FR) maps for each neuron type. The color at each point indicates the average firing rate (in Hz) across neurons in response to tACS with corresponding frequency (horizontal axis) and amplitude (vertical axis). Two points on PLV and FR maps are selected for further analysis: (i) A point with high PLV on Arnold tongue region with tACS parameters of 13.5 Hz and 8 pA. The tACS waveform and membrane potential of a sample PY neuron (top right) show that the PY neuron fires regularly after the peak of the tACS wave (red circles). The phase distribution of tACS wave at firing instances of PY neuron shows a phase preference between 90 and 180 degrees. (ii) A point with low PLV outside of the Arnold tongue region with tACS parameters of 18.5 Hz and 8 pA. The tACS waveform and membrane potential of a sample PY neuron (bottom right) show that the PY neuron fires at random phases of tACS wave (red circles). The phase distribution of the tACS waveform when PY neurons fired shows a uniform phase distribution.

**Fig. 7:**
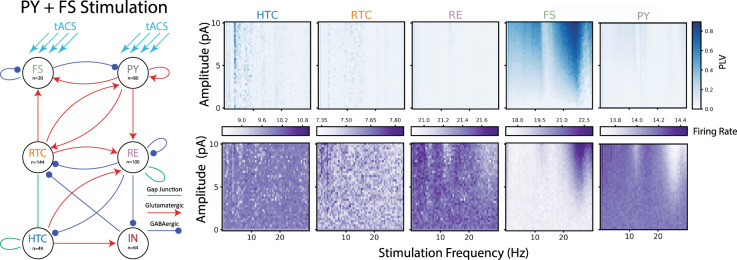
Modeling tACS stimulation to both PY and FS Cells. Phase-locking (top) and firing rate (bottom) response to tACS applied to both the PY and FS cells. Each pixel in the histogram represents five seconds of simulation with the appropriate amplitude tACS stimulation. In the plot, tACS amplitude and frequency range from 0 to 10 pA and 0 to 30 Hz, respectively. The FS neurons exhibit two Arnold tongues, the first centered around the endogenous frequency for the PY neurons, 13.5 Hz, and the second centered at the first harmonic. For PY neurons, an Arnold tongue is also present at the endogenous frequency. Phase-locking for the FS neurons is high compared to the model where FS neurons were not stimulated, especially at the first harmonic. In this plot, the high phase-locking in the FS neurons overshadows the Arnold tongue in the PY neurons, but it is still present.

We adapted and connected two previously developed computational models of a cortical^37^ and thalamic^46^ network for a complementary investigation of the mechanism of action of tACS. The cortical model was composed of fast-spiking inhibitory neurons (FS) and regular-spiking excitatory pyramidal neurons (PY). The thalamic model included excitatory relay-mode thalamo-cortical cells (RTC), excitatory high-threshold bursting thalamic cells (HTC), inhibitory thalamic reticular cells (RE), and inhibitory thalamic interneurons (IN). Synaptic and gap-junction connection patterns between neuronal populations followed previous modeling work (Fig. 6a). Without stimulation, the network reproduced the major oscillatory patterns observed in our experimental LFP recordings from the cortex (PPC) and thalamus (LP) (Fig. 6b). In the model, the simulated LFP displayed dominant alpha (~13.5 Hz) and weak theta-band (~3.6 Hz) oscillations in cortex and dominant theta (~3.6 Hz) and weak alpha (7.3 Hz) in the thalamus (Fig. 6b, right, spectral plots). We next stimulated PY neurons according to the same protocol we used in the experimental investigation of entrainment by tACS. We first demonstrate how simulated tACS with a frequency matching the endogenous alpha oscillation (13.5 Hz) entrains individual neurons and the entire network. The network response to 13.5 Hz and 8 pA stimulation is shown in Fig. 6c, demonstrating the collective entrainment of different neuronal types to stimulation in terms of increased amplitude of cortical LFP compared to its amplitude without stimulation. The membrane potential of a randomly selected neuron from each neuron type in the network shows how individual FS and PY neurons adjust their spike times to tACS. We next applied a comprehensive set of tACS waveforms to cortical PY neurons with frequencies between 3 and 30 Hz and amplitudes between 1 and 10 pA and computed the phase-locking of spikes to tACS for all neuron types (Fig. 6d). We found Arnold tongue patterns (indicated by dark blue shades) centered on the endogenous alpha frequency only for cortical neurons. Compared to cortical neurons, only very weak entrainment was overall observed for thalamic neurons. We observed weak entrainment in the theta frequency, which represents the dominant frequency peak in absence of stimulation. These findings are in agreement with our experimental findings where tACS entrained cortical neurons in PPC, but not thalamic neurons in LP. Interestingly, we found that tACS entrained FS more than PY as indicated by wider and darker Arnold tongue pattern for FS. This behavior agrees with our experimental observations where narrow-spiking neurons of PPC showed greater phase-locking to tACS when compared to broad-spiking neurons. To clarify the neuronal response to tACS at different parts of the PLV heat-map, we further examined the PY response to two tACS waveforms with equal amplitudes, but different frequencies: (i) A point on the Arnold tongue (13.5 Hz, 8 pA), where PY neuron fires regularly after the peak of tACS, with a phase preference between 90 and 180 degrees as shown in phase histogram (Fig. 6d top right), indicating high synchronization by tACS. (ii) A point outside of the Arnold tongue (18.5 Hz, 8 pA), where PY neuron fires at random phases of tACS, without a phase preference (Fig. 6d bottom right), indicating a lack of synchronization by tACS. Finally, we used the biophysical model of thalamo-cortical network to test if the frequency mismatch between dominant thalamic oscillations and alpha tACS was the reason for the absence of Arnold tongues in thalamic records in our experiments. To answer this question, we altered thalamic endogenous oscillations from being theta-dominated to alpha by increasing the DC drive current of HTC neurons from 0 to 50 pA and decreasing the drive current of RTC neurons from 100 to 75 pA. Then we stimulated the pyramidal neurons using the same tACS parameters as we used previously and extracted the phase-locking maps. Indeed, we found that both cortical and thalamic neurons were entrained by tACS and displayed Arnold tongues (Supplementary Fig. S12). We next explored the effect of tACS delivered to both PY and FS cells. We again found that the Arnold tongue governed the interaction between the endogenous oscillation and the applied stimulation waveform. In contrast to the simulation in which only PYs received input, there was a more pronounced Arnold tongue at the first harmonic of the endogenous frequency (Fig. 7). The same pattern was maintained when only FS cells were stimulated (Supplementary Fig. S13). Finally, adding NMDA receptors to the cortical network did not qualitatively change the presence of the Arnold tongue in response to tACS (Supplementary Fig. S14). Together these modeling results provided support for our experimental data that showed stronger phase-locking in narrow spiking neurons when compared to broad-spiking ones in response to tACS.

## Discussion

### tACS entrains spiking activity during endogenous alpha oscillations in awake head-fixed ferrets

We demonstrated the acute effect of tACS on neuronal firing coupled to the endogenous alpha oscillations and thereby provide experimental evidence for the previous predictions derived from computational modeling studies about the mechanism of action of entrainment of ongoing oscillations. We found that tACS entrained the spiking of individual cortical neurons but did not alter the neuronal firing rate. We developed a finite element model of the ferret head, validated the model predictions with experimental measurements of tACS electric potentials in the ferret brain, and tuned the tACS amplitude to produce electric fields comparable to those reported in humans and non-human primates. We found that the phase-locking of spikes by tACS depended on stimulation frequency: matching the frequency of stimulation with endogenous alpha resulted in phase synchronization of spikes regardless of stimulation intensity. For tACS frequencies adjacent to endogenous alpha, higher stimulation intensities were required to achieve similar phase-locking between spikes and tACS. This behavior resulted in a triangular-shaped synchrony region when quantifying the spike-tACS phase-locking as a function of stimulation frequency and amplitude. The triangular region was centered on the endogenous alpha frequency and is known as the Arnold tongue, as previously reported in computational modeling studies of tACS^15,31,37,46,47^. Despite being statistically significant in only a small fraction of neurons, the Arnold tongue was evident in PPC. The Arnold tongue was less developed and less clear in VC, and was absent in the thalamus (LP). Furthermore, we identified that the effect of tACS was cell-type specific. The phase-locking between tACS and spikes was stronger for narrow-spiking neurons compared to their broad-spiking counterparts. This cell-type specificity was further supported by a computational model of thalamo-cortical network with ongoing alpha oscillations (Fig. 6). In the model, the fast-spiking cortical neurons demonstrated higher phase-locking to tACS than broad-spiking neurons.

### Weak electric fields (< 0.5 mV/mm) comparable to tACS field strength in humans and non-human primates can entrain neural spiking

To examine the underlying mechanisms of how tACS modulates oscillatory interactions in the thalamo-cortical network, we first delineated the functional interactions in the PPC-LP-VC network. We identified endogenous alpha oscillations in three regions based on three quantitative measures: LFP spectrum, phase-locking of spikes to population firing rate, and spike-EEG synchrony. Further analysis supported PPC as the source (or driver) of alpha oscillations in the three-node network in resting awake ferrets. These findings of prominent resting-state alpha oscillations in the ferret posterior cortex closely correspond to what has been known for a long time about the dominance of the alpha oscillation in the resting-state EEG in humans^16,48^. Despite this presence of alpha oscillations in all three recording regions and the existing evidence for enhanced phase synchrony by weak electric fields when stimulation and endogenous frequencies are close^17,30,49^, we only found a clear Arnold tongue pattern for PPC neurons. Compared to PPC, VC neurons showed a less-defined triangular region when looking at PLV as a function of stimulation parameters. In addition, the LP neurons did not demonstrate systematic phase-locking to tACS. The magnitude of the tACS electric field (model-driven value) was comparable in all three regions. Thus, the differential field strength is unlikely to be the explanation. Rather, by being the source of alpha oscillations, PPC may respond more strongly to tACS in the alpha frequency. In contradiction to recent reports of failure of electric fields less than 1 mV/mm (resulting from commonly applied tACS intensities) in modulating the dynamics of neuronal circuits^9–11^, the identified PPC Arnold tongue in our work demonstrates how weak electric fields (< 0.5 mV/mm) can engage individual neurons provided that the directionality of information flow and the source of target oscillations are considered when designing the stimulation protocol. Our results demonstrate in vivo how an electric field comparable to (or slightly lower than) the fields predicted and measured in human and non-human primate tACS studies^45^ can result in Arnold tongue regions in synchronization maps.

### The head-fixed awake ferret model to study the mechanism of action of alpha-tACS

Modulation of spiking activity by externally applied subthreshold electric fields has been previously demonstrated in animal studies. Application of a subthreshold (< 10 mV/mm) 20 or 50 Hz oscillating electric field modulated the power and frequency of pharmacologically induced gamma oscillations in CA3 pyramidal cells in rat brain slices^25,29^. Fröhlich and McCormick showed how subthreshold oscillating electric field can enhance slow oscillations recorded from ferret visual cortex slices with effects demonstrated at field amplitudes as low as 0.25 mV/mm^27^. Ozen et al. performed in vivo recordings from anesthetized and behaving rats while applying low intensity slow electric fields (< 1.7 Hz) via electrodes placed on skull or dura, and reported reliable entrainment of neurons^28^. A direct comparison with this study is difficult since it employed voltage and not current stimulation. Ali et al. reported tACS-induced enhancement of slow endogenous oscillations in an anesthetized ferret with stimulation electrodes placed on skull^31^. Despite the fact that these experimental reports present evidence for cellular-level effects of tACS, these studies share three common limitations. First, they have taken advantage of slow-wave oscillations (~ 1 Hz, under anesthesia or in vitro) or fast gamma-band oscillations (~30 Hz, pharmacologically induced, in vitro). Neither of these ongoing oscillations are the best model for the dominant alpha oscillations in the awake brain. Second, using in vitro preparations or animal models with lissencephalic brains imposes limitations on the translation to human tACS. In addition, the way tACS electrodes were configured (bath application in vitro, and direct placement on dura or skull in vivo) does not match with standard practices for tACS electrode placement on the scalp in humans. Third, none of the above-mentioned animal studies have explored the parameters of stimulation in a systematic way to present a comprehensive entrainment map as a function of stimulation frequency and amplitude. Our study overcomes these limitations by taking the advantage of using an awake head-fixed ferret animal model. Ferrets are gyrencephalic and display alpha oscillations in the awake state^42^. This current paper provides acomprehensive demonstration of how the alpha oscillation in the ferret originates in PPC and drives both the thalamus and primary visual cortex, in conceptual agreement with previous findings from studies of non-human primates^41^. Thus, the ferret represents a promising additional model system to investigate tACS and other brain stimulation strategies that target alpha oscillations. In addition, the placement of tACS rubber electrodes on the scalp with conductive gel also made our setting more comparable with commonly used strategies used in human tACS studies.

### Cell-specific effects of tACS

We identified two types of neurons based on their spike waveform shape in PPC and VC: narrow-spiking and broad-spiking neurons. With tACS, the phase of firing was modulated for both cell types, but the effects were more prominent for narrow-spiking neurons in PPC. In VC, despite the lack of a clear Arnold tongue structure, the narrow-spiking neurons also displayed stronger phase-locking to the stimulation waveform. The stronger coupling of narrow-spiking neurons to tACS was consistent with their behavior in absence of tACS (Fig. 2). The narrow-spiking neurons showed stronger coupling to local (population firing rate) and large-scale (EEG) alpha oscillations when compared to broad-spiking neurons. The population ratio of narrow to broad-spiking neurons were 0.81 and 1.46 in PPC and VC, respectively. These ratios differ from the reported excitatory to inhibitory population ratio of 0.25 in cortical regions of mammalian brain^50^. We are unable to make a definite statement about the reason for this mismatch. It could be that more fast-spiking cells are engaged in the alpha oscillation. Or, an alternative explanation could be that axonal spikes get misclassified as fast-spiking units as previously reported for thalamus^51^. However, we did not find a bimodal distribution of spike duration for thalamus, which makes this explanation less likely. Nevertheless, although layer V pyramidal neurons have been suggested to be the direct targets of tACS due to their elongated somato-dendritic axis, our findings suggested that tACS might also modulate the activity of other neuron types including fast-spiking interneurons as previously reported^52–56^. Given that the change in somatic membrane voltage is the largest for pyramidal cell and minimal in spherically symmetrical interneurons, the effects on interneurons are likely the result of their functional role in the targeted network activity and not their morphology^48^. Indeed, in our computational model of thalamo-cortical network that reproduced the key spectral features of our in vivo recordings in ferret PPC and LP, we applied tACS to only pyramidal neurons, and found that both pyramidal and fast-spiking neurons displayed phase-locking to tACS, with fast-spiking neurons showing more phase-locking to tACS (Fig. 6). This supports the notion that the response to tACS can be mediated via direct engagement or indirect network effects that is shaped by the endogenous activity of the cells and their role in the generation of the network activity patterns.

### Limitations

Like all scientific studies, our study has several limitations. First, in the tACS experiments, the phase-locking values for the regions inside the Arnold tongues were small and only slightly higher than the corresponding values for adjacent regions. In addition, only a small number of units (~ 2%) showed significant phase-locking as tested by Rayleigh’s test. We propose that higher synchronization values might be measured if the electrophysiological recordings were acquired from the regions with higher tACS electric field strength as predicted by the computational model of the field distribution in the ferret head. Second, we did not investigate stimulation frequencies in other frequency bands to examine the presence of additional Arnold tongues at the (sub)harmonics^34^. This was an experimental design choice based on time constraints for keeping ferrets comfortably head-fixed. We prioritized the collection of many trials for the stimulation parameters that we examined. In agreement with previous modeling work of tACS^31,37,57^, we did observe an Arnold tongue at the harmonic of the endogenous alpha frequency in our computational model of the thalamo-cortical network. Third, we found comparable electric field magnitudes in the cortex and thalamus for our stimulation montage. Given this lack of focal delivery of electric stimulation by tACS, we cannot exclude that tACS modulated the neural oscillations in other brain areas that we were not able to record from. For example, stimulation may have altered oscillations in other brain areas that in turn modulated the alpha oscillation in PPC. In this study, we have simultaneously recorded activity from three anatomically and functionally interconnected brain areas and found differential effects of the stimulation. Our functional connectivity analysis showed that PPC drives both VC and LP in the alpha-frequency band, and it is thus unlikely that primary stimulation effects in VC and LP are the cause of the modulation by tACS in PPC. To further investigate whether the effect on PPC was due to the electric field induced by tACS directly or an indirect effect from other brain regions, we conducted electric stimulation via bone screws and optogenetics in PPC. Both experiment results support that focal stimulation in PPC modulate the neural oscillations directly. In addition, the amount of space on the scalp to attach the stimulation electrodes was limited to an anterior–posterior setup similar to what has been typically used in human studies^16^. Since the electric field strength was not maximal in these three areas, we are probably underestimating the effect of tACS in our experiments. Furthermore, our results demonstrate that the field strength on its own is not the main determinant of the effect on neuronal networks but rather that endogenous activity patterns and other factors shape the presence and magnitude of neuronal entrainment by tACS. Fourth, our electric field modeling results indicate that the electric field in cortical regions is largely in the posterior–anterior direction, which is perpendicular to the somato-dendritic axis of many pyramidal neurons. Ideally the stimulation electrodes should be placed to maximize the component of the electric field that is normal to ROI cortical surface to increase the field-induced polarization in pyramidal neurons in order to impose stronger phase-locking in target pyramidal neurons. The hardware-imposed constraints of implanted electrodes prevented us from placing tACS electrodes directly above the ROIs, which would have increased the electric field magnitude and component normal to the cortical surface. Future studies should consider the specific geometry of the delivered electric field with respect to the geometry of the targeted neurons and brain areas. Fifth, our simulations of the electric potentials resulting from stimulation closely match up with our experimental measurements. However, we were unable to reliably estimate the local electric field strength within the electrode arrays. This is because, in principle, only two of the three electric field vector components can be estimated with the planar arrangement of the contacts in an electrode array. Further, these estimates are highly sensitive to the specific orientation of the array plane with respect to the local current flow, which cannot be reconstructed accurately. Finally, the electric field components are computed by subtracting the potentials of neighboring contacts and dividing by the distance between the contacts. The small inter-contact distance (0.20 or 0.25 mm) results in large fluctuations of the estimates due to various perturbations in the individual potential recordings, such as from microscopic conductivity conditions at the electrode–tissue interface and small differences in the amplifier gains. Overall, however, our results align with what has been reported in human studies, where more uniform sampling with larger electrode contact spacing (10 mm instead of 100 µm) allowed further validation of electric field amplitude^9^. Sixth, one important question is if tACS elicited phosphenes by direct stimulation of the eye. It is unlikely that the animal experienced phosphenes, but in humans, phosphenes of retinal origin are commonly induced by tACS, especially with frontal electrodes^58–60^. The maximal current density in the eye orbit in our ferret FEM model was 0.103 A/m^2^ with a median of 0.0369 A/m^2^ for the eye closest to the frontal electrode pad in the 80 μA stimulation current condition. Both of these values exceed the estimated threshold for inducing retinal phosphenes of 0.008–0.030 A/m^2^ (0.011–0.043 mV/mm)^60^. Thus, direct retinal stimulation is possible. However, if the reported effects were the results of spurious activation of the visual system by stimulation of the retina or optic nerve, we would expect to see the strongest entrainment effect in VC and not in PPC. Future studies could address this point by training animals to report phosphenes or developing an implant/electrode configuration that minimizes the electric field reaching the orbits. Seventh, tACS can activate nerves in the scalp^59^. This is a possibility in our ferret model as well since we matched the electrode current density to that of human tACS. The threshold for peripheral nerve stimulation with low-frequency waveforms has been estimated to be 3.8–5.8 mV/mm^61^. In our FEM model, the electric field in the scalp for 80 μA current injection reached a maximum of 10.7 mV/mm and exceeded the range of 3.8 mV/mm in a scalp volume of 12.8 mm^3^ in the vicinity of the tACS electrode edges. Thus, stimulation of scalp nerves is a possibility. To address this, we demonstrated in a control experiment that only stimulating peripheral nerves on the animal’s back did not produce the Arnold tongue pattern. This is consistent with Asamoah and colleagues’ work where they showed entrainment in the anesthetized animal for comparable field amplitudes when electrical stimulation was directly applied to the skull^62^, thus demonstrating entrainment by tACS via exclusively cortical stimulation. Eighth, our experimental design was based on the assumption of a fixed endogenous alpha frequency. This was motivated by our observation that individual ferrets display a constant peak frequency that is stable across recording sessions but different between animals. However, fluctuations around this mean peak frequency could have contributed to an underestimation of entrainment since for some stimulation segments the stimulation matched in frequency could have been actually mismatched. We indeed found that the entrainment effect was reduced for stimulation epochs preceding a period where the endogenous alpha frequency did not match the average peak frequency. This numerical effect was present for narrow-spiking cells (*r* = −0.21, *p* = 0.17) but not for the broad-spiking cells (*r* = 0.06, *p* = 0.68), in further agreement with our discovery of a differential role of the two cell types in terms of their response to stimulation. Ninth, our computational biophysical model of the thalamo-cortical network was designed to include the required biophysical complexity to generate alpha oscillations but was not designed to capture all features of the experimental recordings, and was also not tuned to exhibit specific behavior to avoid artificial alignment of computational and biological results due to overfitting. Specifically, our model includes only a single cortical region, in contrast to our experimental recordings from two cortical areas. Our cortical model network consists of only two major cell types and does not capture the richness of different interneuron subtypes. It is also worth pointing out that the entrainment in our computational model is more pronounced than in our experimental data. The main reason for that is that a computational model of this size is too tightly coupled (i.e., too densely connected with too strong synapses) when compared to a real brain. Future work could leverage large-scale simulations that exhibit more plausible levels of endogenous synchronization and thus likely also lower entrainment by tACS.

In conclusion, we found that weak electric fields (< 0.5 mV/mm) can entrain neuronal activity. Of note, the field strength resulting from the stimulation used in our study is comparable to the field strength measured in tACS experiments in humans and non-human primates. In addition, our findings provide in vivo evidence to support the model-driven predictions about how tACS entrains ongoing neuronal oscillations as demonstrated by the Arnold tongue pattern. Our findings underline the importance of considering the endogenous activity patterns, their directionality, and spatial sources for successful entrainment. Our findings re-emphasize the importance of animal models in tACS studies by providing evidence for the effectiveness of weak electric fields in modulating the network oscillations via entraining the activity of neuronal populations.

## Methods

### Animals

Three adult spayed female ferrets (*Mustela putorius furo*, 4 months old at the beginning of the experiment) were used in this study. All animal procedures were performed in compliance with the National Institute of Health guide for the care and use of laboratory animals (NIH publication No. 8023, revised 1987) and the United States Department of Agriculture, and were approved by the Institutional Animal Care and Use Committee of the University of North Carolina at Chapel Hill.

### Head-post, bone screws, and electrode implantation surgery

The initial induction of anesthesia was performed with intramuscular injection of ketamine/xylazine (30 mg/kg of ketamine, 1–2 mg/kg of xylazine). After confirming the loss of paw pinch reflex, animals were intubated for isoflurane (0.5–2% in 100% oxygen) delivery via mechanical ventilation. The physiological parameters including electroencephalogram, partial oxygen concentration, end-tidal CO2, and rectal temperature were continuously monitored throughout the surgical procedure to maintain the animal in a stable state of deep anesthesia. All surgical procedures were performed under sterile conditions. The skull was fixed to a stereotactic frame using a mouthpiece and ear bars to enable accurate identification of target regions and electrode implantations. A custom-designed stainless steel head-post was first secured to the anterior part of the exposed skull via four stainless steel bone screws. A craniotomy was performed over the left hemisphere of PPC to implant Microelectrode arrays in PPC and LP regions. Another craniotomy was performed over the left hemisphere of VC region. The dura and then pia were removed before lowering the LP microelectrode array (2 × 8 tungsten electrodes, 35 µm diameter, 9 mm length, 250 µm spacing; Microprobes for Life Science, Gaithersburg MD). The second microelectrode array (2 × 8 or 2 × 16 tungsten electrodes, 35 µm diameter, 9 mm length, 200 µm spacing; Innovative Neurophysiology, Durham NC) was lowered to deep cortical layers in the lateral gyrus (PPC). The third microelectrode array (2 × 8 tungsten electrodes, 35 µm diameter, 9 mm length, 200 µm spacing; Innovative Neurophysiology) was implanted in VC area after removal of dura and pia layers. The reference electrode was directly adjacent to the corresponding recording electrodes for all three microelectrode arrays. Each microelectrode array had a silver wire for ground connection. Three bone screws on the other hemisphere were used as a ground for each microelectrode array. The EEG bone screws were placed on both sides of the PPC craniotomy. Microelectrode arrays were fixed in place with dental cement. After the dental cement hardened, the muscle and the skin around the incision were sutured together. Animals were administered preventative analgesics and antibiotics for 1 week after surgery while recovering in their home cage for at least one week prior to recordings.

For the animals in the bone-screw stimulation experiments, two bone screws were screwed halfway into the skull, avoiding direct contact with the cortex. The bone screws were positioned just anterior and posterior to the PPC craniotomy, respectively. The subsequent electrode implantation procedures were the same as described above.

### Virus injection and optrode implantation surgery

For the optogenetic stimulation experiment, the preparation and recovery were the same as described above. Before the implantation, 0.4 µL of rAAV5-CaMKII-ChR2-mCherry was injected into PPC. Then, an electrode array that includes an optical fiber (optrode) was implanted (electrode tips and optical fibers are secured 100 and 400 µm above the virus injection site, respectively) (16 channel circular Platinum/Iridium electrode, 125 µm diameter, 5 mm length, 250 µm spacing; fiber optic 4.5 mm length, 200 µm core outer diameter; Microprobes for life science, Gaithersburg, MD). Custom-designed plastic cylinders were implanted around the light fibers to anchor the optical fiber from the laser during recordings and to prevent laser light leakage. Bone screws on the other hemisphere were used as a ground for each microelectrode array. Three weeks elapsed before stimulation experiments for sufficient expression of the opsin in the targeted neurons. Resting-state recordings were collected 1 week after the surgery to identify the individual endogenous alpha frequency. Sinewave modulated optogenetic stimulation was applied around 3 weeks after the surgery.

### Transcranial electric current stimulation procedure

Two circular tACS electrodes (*r* = 5 mm) were prepared by trimming conventional carbon-silicone tACS electrodes. The electrodes were applied to the ferret head using Ten20 conductive EEG paste over the left frontal area (directly above the left eye) and central posterior area (over the neck). The digital tACS waveforms with desired timing and frequency were first generated using a Matlab script, then converted to analog signal using a National Instruments DAQ device. The analog outputs were connected to an A395 linear stimulation isolator (World Precision Instruments) to produce the final tACS currents that were delivered to the head via the tACS electrodes. For each stimulation session, the head-fixed animal received 54 stimulation blocks, each 90 s with 10 s inter-stimulus intervals. Each stimulation block was randomly selected from a pool of 54 different combinations of stimulation amplitudes (5, 10, 15, 20, 40, 80 µA, all zero-to-peak) and frequencies (alpha−4, alpha−3, …, alpha, …, alpha+3, alpha+4 Hz, where alphas stand for the endogenous alpha frequency). This random order was chosen to minimize the theoretical risk of a cumulative effect of stimulation that would bias the results. The stimulation and simultaneous electrophysiological measurements were performed in a room with dim lights while the animal was awake and not engaged in any task.

### Peripheral nerve electric current stimulation procedure

To control for peripheral nerve activation during tACS, the procedure and parameters of the peripheral nerve electric current stimulation were identical to the tACS stimulation procedure with the only difference being the position of the tACS electrodes. Instead of placing the two circular tACS electrodes on the head, in this experiment, the tACS electrodes were placed on the shaved back. The distance between the two electrodes was also kept the same as in the tACS stimulation (i.e., around 6 cm).

### Long-lasting transcranial electric current stimulation procedure

The same tACS setup was used in this procedure. The stimulation was given once per day for 14 days. In each stimulus session, there was a 5 or 10 min of resting-state recording before the onset of tACS (i.e., pre), followed by 20 or 40 min of continuous tACS, and then followed by another 10 minutes of resting-state recording (i.e., post). During the stimulation period, a single frequency (individual endogenous alpha) at 80 μA was applied via the tACS electrodes. The animal was not involved in any other experiment during this long-lasting effect procedure.

### Bone-screw electric current stimulation procedure

To serve as a control for local stimulation, the procedure and parameters of the electric current stimulation were identical to the tACS stimulation procedure with the only difference being the stimulation electrodes. The distance between the two bone screws was about 11 mm, which is closer than for the tACS setup.

### Alternating current-modulated optogenetic stimulation procedure

After about three weeks to allow for virus expression, we started simultaneous optogenetics and electrophysiology recording. A blue laser at 475 nm (Shanghai Laser & Optics Century Co., Ltd., BL473T3) was used to activate the ChR2 in PPC. Before each session, the laser power was calibrated to ensure consistency across sessions, and black sheets were applied as covers around the optic cable connection to avoid light leakage. Laser intensity was modulated by a custom MATLAB script that was used for tACS, with a minor modification for elevating the baseline voltage to ensure that the light was not modulated only for the positive half of the oscillation cycle. We used 19, 20, 21, 22, 25, and 29 mW peak laser power.

### Electric potential measurements

To validate the electric field model of tACS in the ferret brain, we collected intracranial measurements of electric potentials from the electrode arrays in PPC, VC, and LP for one animal across three recording sessions. The potentials of the electrodes in all three ROIs were referenced to a single electrode potential in the proximity of a particular ROI that was changed for each recording session (for simplicity, all sessions were subsequently re-referenced to the first electrode channel of the LP electrode array). During these in vivo measurements, sinusoidal electric current was injected through tACS scalp electrodes using a fixed zero-to-peak current amplitude of 60 or 80 μA. Four blocks of measurements were recorded in an alternating pattern (60, 80, 60, 80 μA); other parameters were kept constant: frequency = 14 Hz, measurement trial duration = 3 min, interval between trials = 24 s. Thus, for each tACS current amplitude there were six recording sets (two blocks and three reference schemes). The electrode potentials were recorded with a sampling frequency of 333.33 Hz, and were passed through a digital band-pass filter (order: 263, generalized equiripple filter with passband of 12–16 Hz, stopband cutoff frequencies = 8, 20 Hz; stopband gain = 90 dB; passband gain = 1 dB). To reliably extract the signal amplitude for each electrode, the recorded potentials were fitted to a reference sinewave using the MATLAB function “fit”. The reference waveform was derived by fitting a sine function to the potential waveforms from the electrode array (LP, PPC, or VC) with the largest signal per recording set. The sine frequency and phase estimates for each available array electrode (*r*^2^ > 0.997) were averaged to define the reference waveform. The amplitude of the reference sinewave was then fitted to the potential from each electrode in each recording set, and data from noisy electrodes (*r*^2^ < 0.75) were discarded. The standard deviation across the recording sets was computed for the remaining fitted amplitudes within each electrode, and the MATLAB “isoutlier” function (with default settings) was used to exclude electrodes with large standard deviation, and therefore poor reproducibility. The amplitude estimates were averaged within the remaining electrodes to reduce measurement noise in the final values. Out of 9 nominally connected electrodes per array, 8, 6, and 8 electrodes survived these quality checks for the LP, PPC, and VC arrays, respectively.

### Computational modeling of electric field distribution in ferret head

We developed a finite element model for a sex- and age-matched ferret head to study the tACS electric field distribution in the brain. The modeling pipeline comprises five steps: image acquisition, image registration, image segmentation, and implant model, complete model meshing, and electric field calculation (Supplementary Fig. S5). CT and MRI images of an in vivo ferret head without any surgical alterations were collected and manually co-registered. These images were segmented into six tissue regions with different electrical properties: white matter, gray matter, cerebrospinal fluid, nasal cavity (air), skull, and scalp. The neck was not modeled since it was outside the MRI field of view. We validated this approximation by attaching a cylinder with scalp conductivity where the neck would be in the ferret model, making the model about 25% longer; the electric field distribution in the brain did not change significantly and the peak field strength decreased by only 1.2%, demonstrating low electric field sensitivity to the inclusion of the neck. The image segmentation was modified to include craniotomy tissue alterations, such as implanted hardware and insulating materials based on CAD models. The three-dimensional model was meshed into finite tetrahedral elements. Using previously reported values^9,63,64^, tissue electric conductivity was assigned to each element of the mesh (Supplementary Table S1). Finally, the finite element problem was solved to calculate the electric potentials and electric field.

### Image acquisition

In vivo magnetic resonance imaging (MRI) of an animal without any surgical alterations was acquired in a Bruker 9.4T scanner (Bruker AVANCE, Billerica, MA). The MR scan was collected using a RARE-sequence, with TR = 2000 ms, TE = 9.610 ms, voxels = isotropic 0.2 mm^3^, FOV = 42 × 42 × 36 mm, 210 sagittal slices. Ex-vivo computed tomography (CT) imaging of the same animal was acquired on an eXplore CT 120 (GE Healthcare) scanner. The CT scan was collected with isotropic 0.0995 mm^3^ voxels and matrix dimension = 430 × 363 × 883. Ex-vivo CT imaging of a different animal with craniotomy was acquired on an eXplore speCZT (GE Healthcare) scanner. The CT scan was collected with isotropic 0.0995 mm^3^ voxels and matrix dimension = 430 × 363 × 883.

### Tissue segmentation

Ferret head model generation began with manual image co-registration of the MRI and CT scans using 3D Slicer (4.8.0)^65^. To correct for bias field inhomogeneity, N4ITKBiasFieldCorrection^66^, a 3D Slicer tool, was applied to the MRI prior to co-registration. This co-registration is able to combine the soft tissues and air seen in the MRI with the hard tissue (skull) seen in the CT. The co-registered images were manually segmented using 3D Slicer into six different regions: white and gray matter, cerebrospinal fluid, nasal cavity (air), skull, and scalp. Further, the three brain ROIs (LP, PPC, and VC) were defined, including both white and gray matter. To avoid problems during meshing of smoothed surfaces derived from the segmentation, we upsampled the segmentation from 430 × 363 × 883 isotropic voxels (0.0995 mm^3^ each) to 538 × 454 × 1104 isotropic voxels (0.0796 mm^3^ each). Smoothing and gap filling of the segmentation were performed by utilizing the interactive segmentation software SimpleWare ScanIP (O-2018.12, SIMPLEWARE Ltd., Exeter, UK).

### Finite element meshing

All hardware affixations were reconstructed using SolidWorks (2016 × 64 Edition) from the registered CT data set, positioned in the model, and converted into segmented regions with the aid of photos taken after surgery unless specified otherwise. The model represented a post-surgery ferret head with craniotomy, implanted hardware components, and insulating materials used in the experimental setup. Images of the electrodes were used in the reconstruction and positioning of realistically shaped tACS electrodes on the model ferret head. The head post, head post bone screws, dental cement, and acrylic glue were represented in the model. The recording electrode arrays were not modeled as they were too small and had a high electrical impedance. The head post was reconstructed and positioned based on post-surgery CT imaging and photos, replacing skull voxels in that region. The head post bone screws were reconstructed using manufacturer datasheets and positioned into the screw holes of the head post, replacing skull voxels in that region. The acrylic glue was reconstructed with a rectangular prism shape that was modified to wrap around the head post and the head of the bone screws by subtracting skull, head post, and bone-screw voxels from it. The dental cement filling the craniotomy was reconstructed by merging a cylinder and a rectangular prism shape and wrapping the composite around the head post and dental cement. The shape replaced scalp voxels in that region and was subtracted from the skull, head post, and acrylic glue voxels. To prevent overlapping, all tissues and materials were subtracted from each other and combined together from the inside out. The ScanFE module in ScanIP was used to convert the segmented images of both head models to finite element meshes. The resulting mesh consisted of 4.2 million tetrahedrons with 0.74 million nodes.

### Electric potential and field computation

The electric potentials, electric field, and current density were computed in COMSOL Multiphysics 5.4 and 5.5 (COMSOL Inc., Burlington, MA, USA). The finite element mesh was imported into COMSOL, and a set of isotropic conductivity values were assigned to the mesh as listed in Supplementary Table S1. The top surface of the occipital stimulation pad was injected with a fixed current of 60 or 80 μA, while the top surface of the frontal stimulation pad was assigned as ground. To calculate the electric potential and field distribution, the quasi-static Laplace equation

1 $$\nabla .\left(\sigma \nabla V\right)=0$$

was solved with appropriate boundary conditions using the preconditioned conjugate solver and relative tolerance of 10^−6^, where *V* and *σ* represent the electric potential and electric conductivity, respectively. Using the quasi-static approximation makes it possible to scale the stimulation current linearly to achieve the desired electric potential or electric field. The electric potentials were extracted from specific coordinate points in the model corresponding to the ideal ROI electrode locations, as the ROI electrodes were assumed to be small point-like electrodes.

Figure 4a–c and f–j were plotted in MATLAB R2019a. Figure 4d, e and Supplementary Fig. S7 were plotted in COMSOL Multiphysics 5.4/5.5. Supplementary Fig. S6 was created using MATLAB R2019a and visualized in SCIRun 4.7 (R45839) after exporting the finite element mesh and the current density at the center of each tetrahedral element from COMSOL and converting it to electric field strength using respective tissue conductivities. To extract the surface-normal electric field component, first outward-pointing normal vectors of unit length were computed for each brain-surface triangle as averages of surrounding triangles. SCIRun was used to linearly interpolate the electric field in the gray matter onto the surface-nodes, and the dot product of the field and the nodal surface normal was taken to compute the surface-normal electric field component.

### Verifying electrode positions with histology

The animals were euthanized with an overdose of ketamine/xylazine after reaching the scientific endpoint and perfused immediately with 4% phosphate-buffered paraformaldehyde. After removing the brain from the skull, it was post-fixed overnight in the same solution, cryoprotected in 30% phosphate-buffered sucrose solution, shock frozen in dry ice, and cut into 50-µm-thick sections with a cryostat (CM3050S, Leica Microsystems). Sections were separated into two series and stained for cells (Nissl) or cytochrome oxidase. Imaging was conducted with an Aperio VERSA bright-field slide scanner at ×10 magnification. The electrode arrays were located in the sections by electrode tracks, tissue damage or loss, and atlas-based reconstructed by comparing and documenting the sections to ferret atlas plates^67^.

### Electrophysiological data analysis

#### Spectral-domain analysis

We used wavelet transform for spectral decomposition. The wavelets $(w(t,{f}_{0}))$ have Gaussian shape both in time and frequency:

2 $$w\left(t,{f}_{0}\right)=A\cdot {{exp }}\left(-{t}^{2}/2{{{{\sigma }}}_{t}}^{2}\right)\cdot {\rm{exp }}\left(-2i\pi {f}_{0}t\right)$$

where ${f}_{0}$ is the central frequency, $A={({{\rm{\sigma }}}_{t}\sqrt{\pi })}^{-1/2}$ is a normalization factor to make the wavelet energy equal to 1, and ${{\rm{\sigma }}}_{t}$ is the time domain standard deviation that relates to frequency domain standard deviation (${{\rm{\sigma }}}_{f}$) as

3 $${{\rm{\sigma }}}_{t}=\frac{1}{2\pi {{\rm{\sigma }}}_{f}}$$

The frequency-domain standard deviation is defined as a constant depending on the central frequency of the wavelet as *σ*_*f*_ = *f*_0_/7 ^68^. The local field potential or EEG signal was then convolved with Morlet’s wavelets to produce the complex-value analytical signal $X\left(t,{f}_{0}\right)$ at each frequency of interest. We used a family of 100 Morlet wavelets with central frequencies logarithmically spaced between 2 and 100 Hz. Then, the power spectrum of the signal was computed by squaring the absolute value of an analytical signal. For a given region of interest, session, and animal, the power spectrum traces corresponding to all channels were plotted collectively to visually identify and exclude noisy channels. For each animal and for each region of interest we computed the average power spectrum as the mean spectrum across channels and then across sessions.

#### Spike extraction, sorting, and clustering

LFP signals were first high-pass filtered (300 Hz, 4th order Butterworth filter). We determined the filter order by comparing the spectral contents of a filtered signal using different filter orders to fully suppress the stimulation. The spiking threshold was set to negative four times the standard deviation of the high-passed filtered signal, to extract spike times and spike waveforms. The extracted spikes were automatically sorted based on a template matching method implemented in Kilosort^69^, followed by an automatic post-hoc merging, and finally a manual curation by merging the automatically assigned spike groups. Next, we used spike half-width to perform k-means clustering of neurons to two putative subgroups of narrow- and broad-spiking neurons^70^. Increasing the number of clusters did not improve the clustering performance as measured by within-cluster sums of point to centroid distances. Other spike shape features (trough-to-peak time, peak value of normalized spikes, upward slope, spike energy, absolute peak-to-trough ratio, repolarization area, spike minimum amplitude, spike maximum amplitude) did not have multi-modal distributions and were not used in spike clustering.

#### Phase-locking value (PLV)

The consistency of phase differences between two signals is indicative of existing phase synchrony and can be measured by PLV^71^. We computed the PLV to quantify the phase synchrony between two LFP signals or the phase synchrony between individual spikes and tACS waveform. According to the instantaneous phase of tACS waveform at a specific carrier frequency (${f}_{c}$), we assigned a phase to every spike of a single unit, and built a phase distribution for each unit. Then we computed the PLV of each unit with N spikes at a carrier frequency of ${f}_{c}$defined by the following formula:

4 $${\rm{PLV}}\left({f}_{c}\right)=\frac{1}{N}\left|\mathop{\sum }\limits_{n=1}^{N}{e}^{i\left({\theta }_{n}\left({f}_{c}\right)\right)}\right|$$

where ${\theta }_{n}({f}_{c})$ is the instantaneous phase at frequency ${f}_{c}$ assigned to a spike. We used a fixed number of $N=200$ randomly selected spikes per unit, computed PLV, and repeated for each unit 200 times, with the mean PLV across all permutations as the final result. We assessed the significance of modulation of spike phases by tACS using Rayleigh’s test of uniformity, controlled for the number of spikes^72^. The PLV between spikes and LFP was computed in a similar way with the exception that the spike phase was determined using LFP phases, and a fixed number of 1000 spikes were used to extract the PLV. The PLV between two LFP signals was computed in a similar way as used for computation PLV for spike-tACS, with the exception that in this case ${\theta }_{n}({f}_{c})$ was the difference between instantaneous phases of two LFP signals.

#### Directed functional connectivity

The Wiener–Granger causality algorithm^73–75^ infers the influence of a process *y* on a process *x* by building two autoregressive models: a full model where previous values of both *x* and *y* processes are used to estimate the current value of *x*, and a reduced model where the prediction of current *x* value is made solely based on previous *x* values. The algorithm then compares corresponding residuals from two models and infers the directionality from *y* to *x* if the full model results in a less residual error. We used the MVGC toolbox^76^ to apply a conditional form of Wiener–Granger causality analysis in the frequency domain to study the directionality of different oscillatory frequencies in a network of three nodes at PPC, VC, and LP. The MVGC toolbox models the observed data using a vector autoregressive (VAR) approach without assuming a linear scheme for observed real data. The process starts with estimating the model order in the MVGC toolbox using Akaike or Bayesian information criteria or cross-validation. The next step is to estimate the parameters of the VAR model for both the full model (considering the effects of both time series) and the reduced model (considering only one time series). Finally, the Granger causality measure was estimated based on the estimators of residuals of covariance matrices. We performed directionality analysis on LFP time series downsampled to 200 Hz, then windowed into segments of 10 s length (with 75% overlap). LFP time series were collected from 10 randomly selected channels from each region, the directionality analysis was performed on all possible pairs, then averaged across to produce final results for PPC–VC, PPC–LP, and VC–LP pairs. We used the Akaike method for model order estimation with a maximum allowed model order of 20.

#### Spike-triggered population firing rate and spike-triggered EEG

We computed the population firing rate function for each region of interest using spike times collected from that region via multiple channels of the implanted microelectrode array. For each region, the spike times from all channels were pooled together, then sorted, then a binary time series were constructed based on sorted collective spike times with an assigned value of one at time of spikes occurrence, and zero otherwise. We next convolved the binary time series with a Gaussian kernel with a standard deviation of $c=4.6$ ms and kernel width of 30 ms. We constructed the spike-triggered population firing rate and spike-triggered EEG by extracting a 1-s window of population firing rate or EEG around each spike, then averaging across spikes.

### Computational modeling of thalamo-cortical network

We adopted our previously developed computational models of cortex^37^ and thalamus^46^ and connected them in a biologically plausible way^77^ to form the unified thalamo-cortical (TC) model. In short, the cortical part includes 80 pyramidal (PY) and 20 fast-spiking inhibitory (FS) neurons, spatially arranged on a line. Connections within PY neurons are global with a connection probability of 0.5. Each FS was connected to 10 adjacent FS neurons with a probability of 0.8. The reciprocal PY-FS connection was local where every FS was randomly connected to 0.8 of 32 surrounding PY. A biophysical synaptic model based on *α*-amino-3hydroxy-5-isoxazolepropionic acid (AMPA) or γ-aminobutyric acid type A (GABAA)-mediated synaptic currents were used to connect cell populations. Individual PY and FS cells were modeled using Izhikevich formalism for point neurons^78^ with the same parameter values reported previously^37^. The thalamic network included 144 relay-model thalamic cells (RTC), 100 reticular inhibitory neurons (RE), 49 high-threshold bursting thalamic cells (HTC), and 64 local interneurons (IN), all placed in a two-dimensional grid. All chemical synaptic connections were global in the thalamic model with a connection probability of 0.3 for HTC → IN, IN → RTC, and 0.2 for HTC → RE, RTC → RE, RE → HTC, RE → RTC, and RE → RE. All gap-junction connections were local, with a connection probability of 0.3. All thalamic neurons were modeled using Hodgkin–Huxley formalism of point neurons, connected with glutamatergic (mediated by both AMPA and NMDA receptors) and GABAergic (mediated by GABAA receptors) synaptic currents (with implemented short-term synaptic depression), or via gap junctions, with parameter values described previously^46^. The synthetic local field potential (LFP) for the cortex was modeled as the average sum of the absolute values of excitatory and inhibitory currents entering PY neurons^79^. The thalamic LFP was obtained from the average membrane potential of neurons. The tACS was modeled as a current injection into PY neurons. The stimulation amplitude was systemically varied up to 10 pA, which caused a 2 mV change in membrane voltage in an isolated model neuron. Given that an electric field of 2 V/m has been found to cause an ~0.5 mV polarization of the membrane voltage^27^, the lower amplitude values in our simulations span the range of human tACS studies, whereas the upper values are of relevance for a more complete picture of the response dynamics and for future studies employing higher stimulation amplitudes. The computational modeling of thalamo-cortical network was implemented using the Brian2 simulator in Python^80^.

Our model was implemented by connecting a thalamic model, constructed exactly as previously outlined^46^, to a cortical model, constructed as previously described in detail^37^ with some minor changes in parameter values. Changes to the parameter values are noted in the following brief description of the model equations. Full parameter definitions and values have been reported both for the thalamic^46^ parameters and the cortical model^37^.

#### Thalamic model

*Current balance*:

5 $${C}_{\mathrm{m}}\frac{{{\mathrm{d}}V}}{{{\mathrm{d}}t}}=-{g}_{\mathrm{L}}\left(V-{E}_{\mathrm{L}}\right)-{g}_{{\mathrm{KL}}}\left(V-{E}_{{\mathrm{KL}}}\right)-\sum {I}^{{\mathrm{int}}}-\sum {I}^{{\mathrm{syn}}}+{I}_{{\mathrm{app}}}$$

*Ion currents*:

6 $${I}_{i}={{g}_{i}{m}^{p}{h}^{q}\left(V-{E}_{i}\right)}^{\ast }$$

*I_CAN_ current uses modified equation. See Li et. al.^57^ for details.

*Maximal conductance densities g*_*i*_ *(mS/cm*^*2*^*) of ionic currents*:

**Table:** 

	*I*_Na_	*I*_DR_	*I*_H_	*I*_Ca/L_	*I*_Ca/T_	*I*_Ca/HT_	*I*_AHP_	*I*_CAN_
HTC	90	10	0.01	0.5	2.1	3.0	0.3	0.5
RTC	90	10	0.01	0.3	2.1	0.6	0.1	0.6
IN	90	10	0.05	–	–	2.5	0.2	0.1
RE	90	10	–	–	1.3	–	0.2	0.2

*Gating variable (m or h) kinetic equations*:

7 $$\frac{{{\mathrm{d}}x}}{{{\mathrm{d}}t}}={\varphi }_{x}\frac{{x}_{{{\infty }}}\left(V,{\left[{\rm{Ca}}\right]}_{i}\right)-x}{{\tau }_{x}\left(V,{\left[{\rm{Ca}}\right]}_{i}\right)}$$

(Or in an equivalent form below)

8 $$\frac{{{\mathrm{d}}x}}{{{\mathrm{d}}t}}={\phi }_{x}\left({\alpha }_{x}\left(V,{\left[{\rm{Ca}}\right]}_{i}\right)\left(1-x\right)-{\beta }_{x}\left(V,{\left[{\rm{Ca}}\right]}_{i}\right)x\right)$$

*Kinetic parameters of all gating variables*:

**Table:** 

Current type	Gating variable	${\phi }_{x}$	*α*_*x*_ or *x*_∞_	*β*_*x*_ or *τ*_*x*_ (ms)
*I*_Na_	*p* = 3	1	${\alpha }_{{m}}=\frac{0.32(V-{V}_{{{SH}}}-13)}{1-{\rm{exp}} (-(V-{V}_{{{SH}}}-13)/4)}$ *V*_SH_ = −30 for HTC and IN *V*_SH_ = −40 for RTC and RE	${\beta }_{{\mathrm{m}}}=\frac{-0.28(V-{V}_{{\mathrm{SH}}}-40)}{1-{\rm{exp}} ((V-{V}_{{\mathrm{SH}}}-40)/5)}$
*I*_Na_	*q* = 1	1	${\alpha }_{\mathrm{h}}=0.128{\rm{exp}} (-(V-{V}_{\mathrm{SH}}-17)/18)$	${\beta }_{\mathrm{h}}=\frac{4}{1+{\rm{exp}} (-(V-{V}_{\mathrm{SH}}-40)/5)}$
*I*_DR_	*p* = 4	0.25 (RE: 1)	${\alpha }_{\mathrm{m}}=\frac{0.032(V-{V}_{\mathrm{SH}}-15)}{1-{\rm{exp}} (-(V-{V}_{\mathrm{SH}}-15)/5)}$	${\beta }_{\mathrm{m}}=0.5{\rm{exp}} (-(V-V{}_{\mathrm{SH}}-10)/40)$
*I*_H_	*p* = 1	1	${m}_{\infty }=\frac{1}{1+{\rm{exp}} ((V+75)/5.5)}$	${\tau }_{\mathrm{m}}=\frac{1}{{\rm{exp}} (-0.086V-14.59)+{\rm{exp}} (0.0701V-1.87)}$
*I*_Ca/L_	*p* = 2	4.6	${m}_{\infty }=\frac{1}{1+{\rm{exp}} (-(V+10)/4)}$	${\tau }_{\mathrm{m}}=0.4+\frac{0.7}{{\rm{exp}} (-(V+5)/15)+{\rm{exp}} \left.((V+5)/15)\right)}$
*I*_Ca/L_	*q* = 1	3.7	${h}_{\infty }=\frac{1}{1+{\rm{exp}} ((V+25)/2)}$	${\tau }_{\mathrm{h}}=300+\frac{100}{{\rm{exp}} (-(V+40)/9.5)+{\rm{exp}} \left.((V+40)/9.5)\right)}$
*I*_Ca/T_	*p* = 2	4.6	${m}_{\infty }=\frac{1}{1+{\rm{exp}} (-(V+62)/6.2)}$	${\tau }_{\mathrm{m}}=0.612+\frac{1}{{\rm{exp}} (-(V+135)/16.7)+{\rm{exp}} \left.((V+19.8)/18.2)\right)}$
*I*_Ca/T_	*q* = 1	3.7	${h}_{\infty }=\frac{1}{1+{\rm{exp}} ((V+86)/4)}$	When *V* < −83 mV, ${\tau }_{\mathrm{h}}=\exp ((V+470)/66.6)$ When *V* ≥ −83 mV, ${\tau }_{\mathrm{h}}=\exp (-(V+25)/10.5)+28$
*I*_Ca/HT_	*p* = 2	4.6	${m}_{\infty }=\frac{1}{1+\exp (-(V+34)/6.2)}$	${\tau }_{\mathrm{m}}=0.612+\frac{1}{\exp (-(V+107)/16.7)+\exp \left.((V-8.2)/18.2)\right)}$
*I*_Ca/HT_	*q* = 1	3.7	${h}_{\infty }=\frac{1}{1+\exp ((V+58)/4)}$	When *V* < −55 mV, ${\tau }_{\mathrm{h}}=\exp ((V+442)/66.6)$ When *V* ≥ −55 mV, ${\tau }_{\mathrm{h}}=\exp (-(V-3)/10.5)+28$
*I*_Ca/T_ (RE)	*p* = 2	6.9	${m}_{\infty }=\frac{1}{1+\exp (-(V+55)/7.4)}$	${\tau }_{\mathrm{m}}=3+\frac{1}{\exp ((V+30)/10)+\exp \left.(-(V+105)/15)\right)}$
*I*_Ca/T_ (RE)	*q* = 1	3.7	${h}_{\infty }=\frac{1}{1+\exp ((V+83)/5)}$	${\tau }_{\mathrm{m}}=85+\frac{1}{\exp ((V+51)/4)+\exp \left.(-(V+410)/50)\right)}$
*I*_CAN_	*p* = 1	1	${m}_{\infty }=\frac{1}{1+\exp (-(V+43)/5.2)}$	${\tau }_{\mathrm{m}}=1.6+\frac{2.7}{\exp (-(V+55)/15)+\exp \left.((V+55)/15)\right)}$
*I*_AHP_	*p* = 1	1	${m}_{\infty }=\frac{48\cdot {{[{\rm{Ca}}]}^{2}}_{i}}{48\cdot {{\left[{\rm{Ca}}\right]}^{2}}_{i}+0.09}$	${\tau }_{\mathrm{m}}=\frac{1}{48\cdot {{\left[{\rm{Ca}}\right]}^{2}}_{i}+0.09}$

*Calcium dynamics*:

9 $$\frac{{\mathrm{d}}{\left[{{\rm{Ca}}}^{2+}\right]}_{i}}{{{\mathrm{d}}t}}=-\frac{{I}_{{\rm{Ca}}}}{{zFw}}+\frac{{\left[{{\rm{Ca}}}^{2+}\right]}_{{{\mathrm{rest}}}}-{\left[{{\rm{Ca}}}^{2+}\right]}_{i}}{{\tau }_{{{\mathrm{Ca}}}}}$$

*Gap junction*:

10 $${I}_{{{\mathrm{gap}}}}=\frac{{V}_{{{\mathrm{post}}}}-{V}_{{{\mathrm{pre}}}}}{{R}_{{\mathrm{g}}}}$$

*Synaptic current*:

11 $${I}_{{{\mathrm{syn}}}}={sD}{g}_{{{\mathrm{syn}}}}B\left(V\right)(V-{E}_{{{\mathrm{syn}}}})$$

*Gating variable*:

12 $$\frac{{{\mathrm{d}}s}}{{{\mathrm{d}}t}}=\alpha \left[T\right]\left(1-s\right)-\beta s$$

*Magnesium block for NMDA synapses*:

13 $$B\left(V\right)={1/\left(1+{\rm{exp }}\left(-\frac{\left(V+25\right)}{12.5}\right)\right)}^{\ast \ast }$$

**For GABA_A_ and AMPA, $B\left(V\right)=1.$

*Short-term synaptic depression*:

14 $$D=1-\left(1-{D}_{i}(1-U)\right){\rm{exp }}\left(-\frac{t-{t}_{i}}{\tau }\right)$$

See Li et. al.^57^ for full details.

#### Cortical model

*Current balance (PY)*:

15 $$100\frac{{{\mathrm{d}}v}}{{{\mathrm{d}}t}}=0.7\left(v+60\right)\left(v+40\right)-u+I$$

16 $$\frac{{{\mathrm{d}}u}}{{{\mathrm{d}}t}}=0.03\left[-2\left(v+60\right)-u\right]$$

17 $$I={I}_{{\rm{dc}}}+{I}_{{\rm{syn}}}+{I}_{{\rm{stim}}}+{I}_{{\rm{noise}}}$$

$${\rm{reset}}\; {\rm{rule}}:\; {\rm{if}}\;v\ge +35,{\rm{then}}\;v\leftarrow -50,u\leftarrow u+100.$$

*Current balance (FS)*:

18 $$20\frac{{{\mathrm{d}}v}}{{{\mathrm{d}}t}}=\left(v+55\right)\left(v+40\right)-u+I$$

19 $$\frac{{{\mathrm{d}}u}}{{{\mathrm{d}}t}}=0.2\left\{U\left(v\right)-u\right\},\qquad U\left(v\right)=\left\{\begin{array}{c}0\;\;\;\;\;\;\;\;\;\;\;\;\;\;\;\;\;\;\;\;\;\;{\rm{when}}\;v\le {v}_{b}\\ \!\!\!0.025{\left(v+55\right)}^{3}\;\;{\rm{otherwise}}\end{array}\right.$$

20 $$I={I}_{{\rm{dc}}}+{I}_{{\rm{syn}}}+{I}_{{\rm{stim}}}+{I}_{{\rm{noise}}}$$

$${\rm{reset}}\; {\rm{rule:}}\; {\rm{if}}\;\;v\ge +25,\;{\rm{then}}\;\;v\leftarrow -45$$

*Synapses*:

21 $${I}_{\rm{syn}}=g(t)B\left({V}_{\rm{m}}\right)\left({V}_{\rm{m}}-{E}_{\rm{rev}}\right)$$

22 $$g(t)={g}_{{t}_{\rm{spike}}}{\rm{exp}}(-t/\tau)$$

23 $${\rm{Upon}}\; {\rm{presynaptic}}\; {\rm{action}}\; {\rm{potential:}}\;{g}_{{t}_{{{\mathrm{spike}}}}}\leftarrow g+{g}_{{\max }}$$

*Magnesium block for NMDA synapses*:

24 $$B\left({V}_{\rm{m}}\right)={\frac{{\left(\frac{{V}_{\rm{m}}+80}{60}\right)}^{2}}{1+{\left(\frac{{V}_{\rm{m}}+80}{60}\right)}^{2}}}^{\ast\ast }$$

**For GABA_A_ and AMPA, $B\left({V}_{\rm{m}}\right)=1.$

*Cortical LFP measured from PY neurons*:

25 $${\rm{LFP}}=\mathop{\sum }\limits_{i=1}^{N}\frac{\left|{I}_{{\rm{AMPA}},i}\right|+\left|{I}_{{\rm{NMDA}},i}\right|+\left|{I}_{{{\rm{GABA}}}_{{\rm{A}}},i}\right|}{N},N{\rm{:number}}\; {\rm{of}}\; {\rm{PY}}\; {\rm{neurons}}$$

*Addition of NMDA synapses to the cortical model*:

For experiments with NMDA synapses in the cortical model (Supplementary Fig. S14), NMDA synapses were added to existing PY → PY glutamatergic connections. NMDA synapse parameters were set to ${g}_{{\max }}=0.015$ nS, $\tau =150$ mS, and ${E}_{{{\rm{rev}}}}=0$ mV ^76^. The conductance increase ${g}_{{\max }}$ for RTC → PY AMPA synapses was reduced from 0.3 to 0.1 nS and direct current stimulation to PY and FS cells was adjusted to ${I}_{{\rm{dc}}}=75$ pA for PY cells, and ${I}_{{\rm{dc}}}=58$ pA for FS cells.

### Statistical analysis

The comparison of means for two populations was performed using an independent *t*-test with significance levels at 0.05 or 0.01. The significance of phase-locking values was assessed by the Rayleigh test for non-uniformity of circular distribution of phases.

### Reporting summary

Further information on research design is available in the Nature Research Reporting Summary linked to this article.

## Supplementary information

Supplementary Information

Reporting Summary

## Data Availability

Due to the large file size, the data sets generated and analyzed during the current study are available from the corresponding author upon request. Source data are provided with this paper.

## Source data

Source Data

## Code availability

Custom Matlab code that was used to analyze is available from the corresponding author upon request.
