# Supplementary material for: Transcranial alternating current stimulation entrains alpha oscillations by preferential phase synchronization of fast-spiking cortical neurons to stimulation waveform
Source: Nat Commun. 2021 May 25;12:3151. doi: 10.1038/s41467-021-23021-2 (PMC8149416; doi:10.1038/s41467-021-23021-2)
Supplement: Supplementary file 1 — Supplementary Information [file 41467_2021_23021_MOESM1_ESM.pdf]

SUPPLEMENTARY INFORMATION

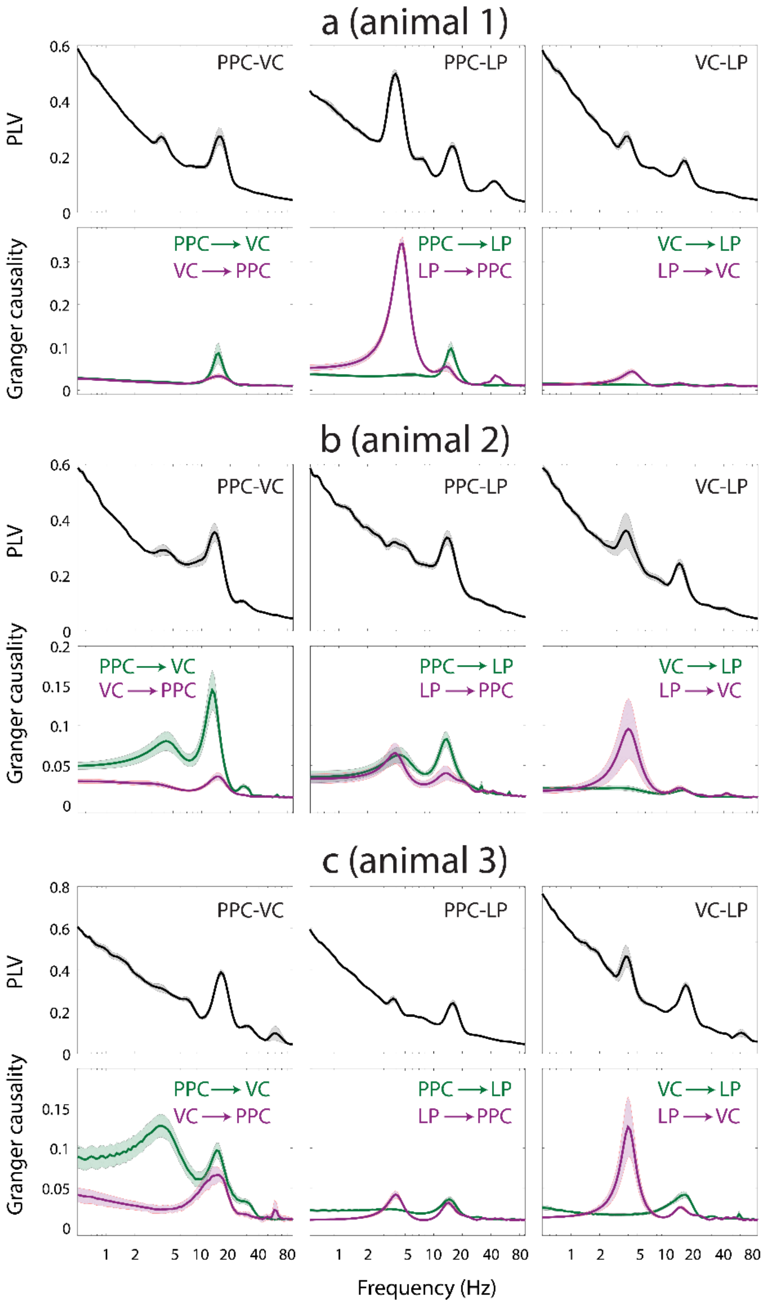

Supplementary Figure S1 Synchronization between regions and directionality analysis for individual animals. For each animal, the top row shows the frequency dependent phase-locking values (PLV), and the bottom row displays the spectrally resolved conditional Granger causality results. The scales for PLV and GC were chosen to facilitate comparisons between regions within each animal. Different animals might have different ranges of values due to individual differences, and the goal here was not to compare between animals. Related to Figure 3. All measures are shown as mean (thick line) ± SEM (light background). N=12, 13, 12 sessions for animal 1, 2, 3 respectively.

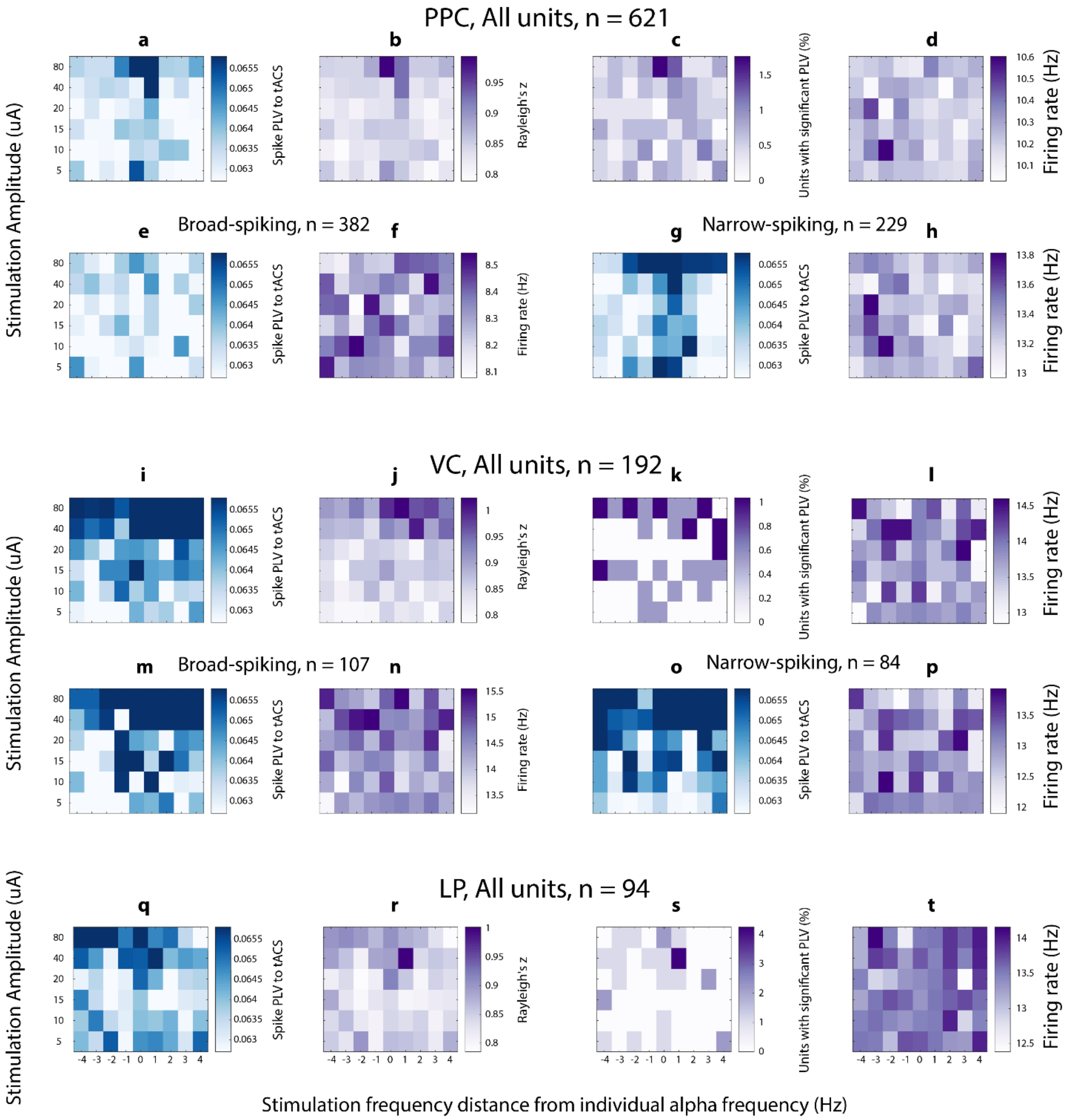

Supplementary Figure S2 Synchronization and firing rate maps for animal 1.

a) Average phase locking of PPC spikes (averaged across 621 units) to tACS phase as measured by phase-locking value (PLV) as a function of stimulation frequency (horizontal axis) and stimulation amplitude (vertical axis).

b) Average Rayleigh's z-score (across all PPC units) as a function of stimulation parameters.

c) Percentage of PPC units with significant PLV as indicated by Rayleigh's test.

d) Average firing rate of all PPC units.

e) Average PLV of broad-spiking PPC units (n=382).

f) Average firing rate of broad-spiking PPC units.

g) Average PLV of narrow-spiking PPC units (n=229).

h) Average firing rate of narrow-spiking PPC units.

i-p) Corresponding measures of (a-h) for VC.

q-t) Corresponding measures of (a-d) for LP. The horizontal axis shows the distance from endogenous alpha frequency in Hz. Related to Figure 5.

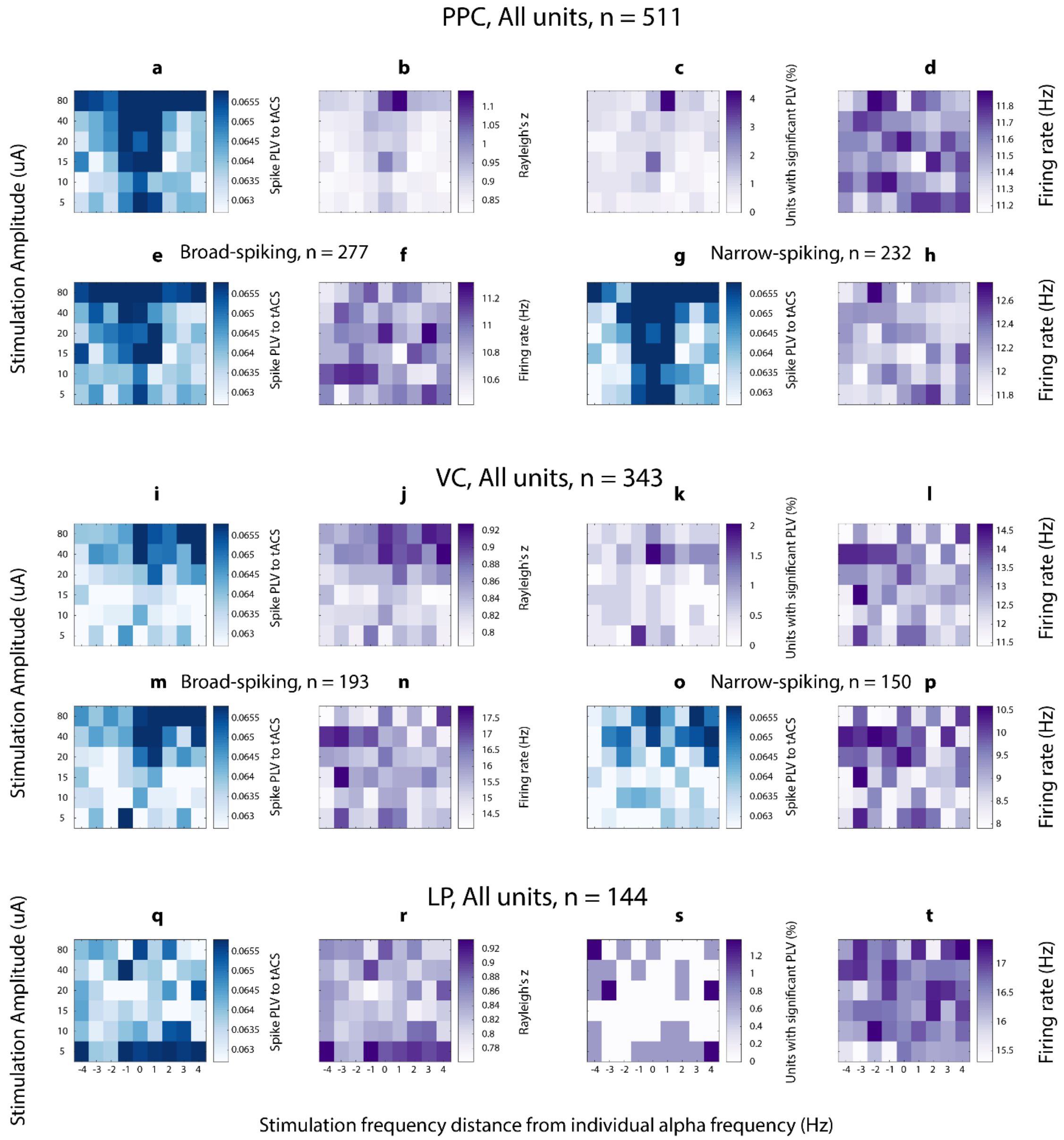

Supplementary Figure S3 Synchronization and firing rate maps for animal 2.

a) Average phase locking of PPC spikes (averaged across 621 units) to tACS phase as measured by phase-locking value (PLV) as a function of stimulation frequency (horizontal axis) and stimulation amplitude (vertical axis).

b) Average Rayleigh's z-score (across all PPC units) as a function of stimulation parameters.

c) Percentage of PPC units with significant PLV as indicated by Rayleigh's test.

d) Average firing rate of all PPC units.

e) Average PLV of broad-spiking PPC units (n=382).

f) Average firing rate of broad-spiking PPC units.

g) Average PLV of narrow-spiking PPC units (n=229).

h) Average firing rate of narrow-spiking PPC units.

i-p) Corresponding measures of (a-h) for VC.

q-t) Corresponding measures of (a-d) for LP. The horizontal axis shows the distance from endogenous alpha frequency in Hz. Related to Figure 5.

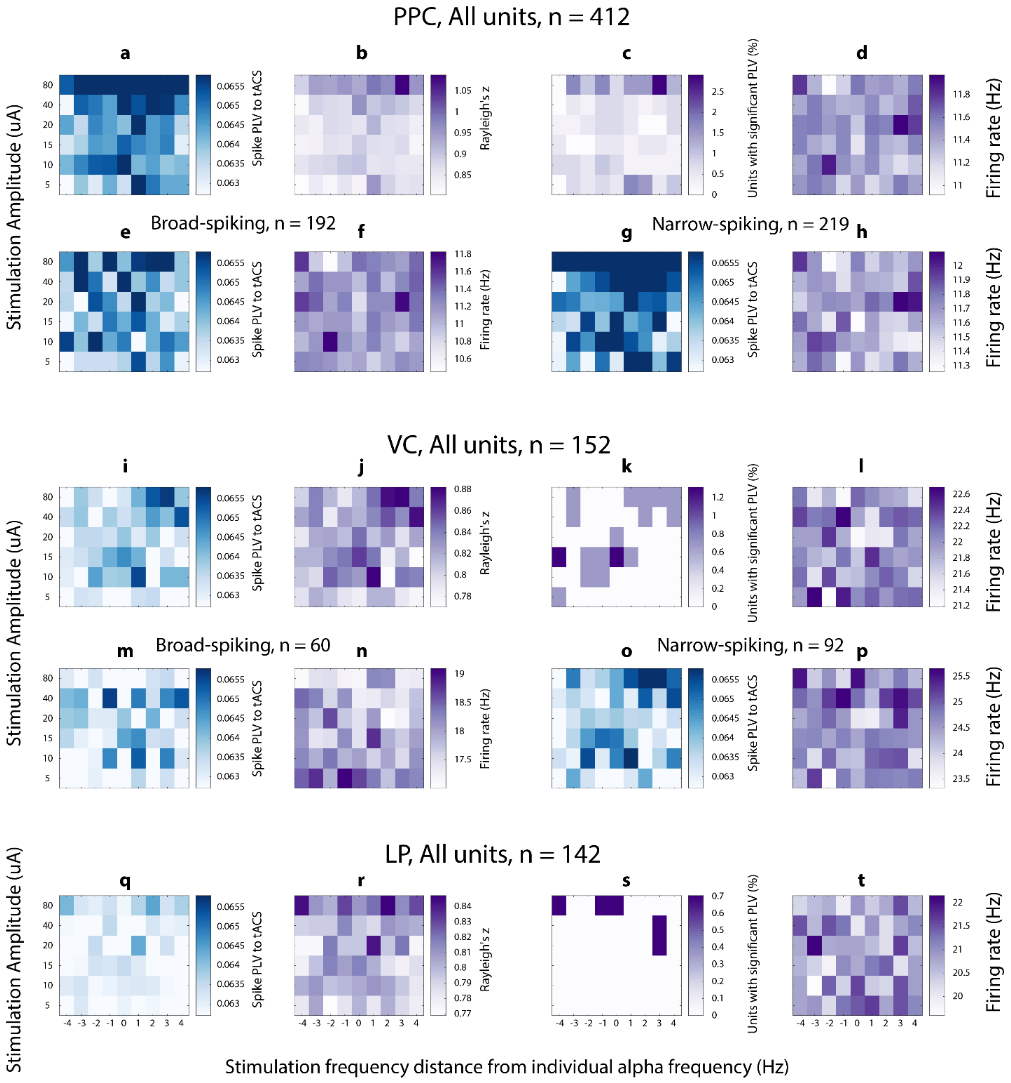

Supplementary Figure S4 Synchronization and firing rate maps for animal 3.

a) Average phase locking of PPC spikes (averaged across 621 units) to tACS phase as measured by phase-locking value (PLV) as a function of stimulation frequency (horizontal axis) and stimulation amplitude (vertical axis).
b) Average Rayleigh's z-score (across all PPC units) as a function of stimulation parameters.
c) Percentage of PPC units with significant PLV as indicated by Rayleigh's test.
d) Average firing rate of all PPC units.
e) Average PLV of broad-spiking PPC units (n=382).
f) Average firing rate of broad-spiking PPC units.
g) Average PLV of narrow-spiking PPC units (n=229).
h) Average firing rate of narrow-spiking PPC units.

i-p) Corresponding measures of (a-h) for VC.

q-t) Corresponding measures of (a-d) for LP. The horizontal axis shows the distance from endogenous alpha frequency in Hz. Related to Figure 5.

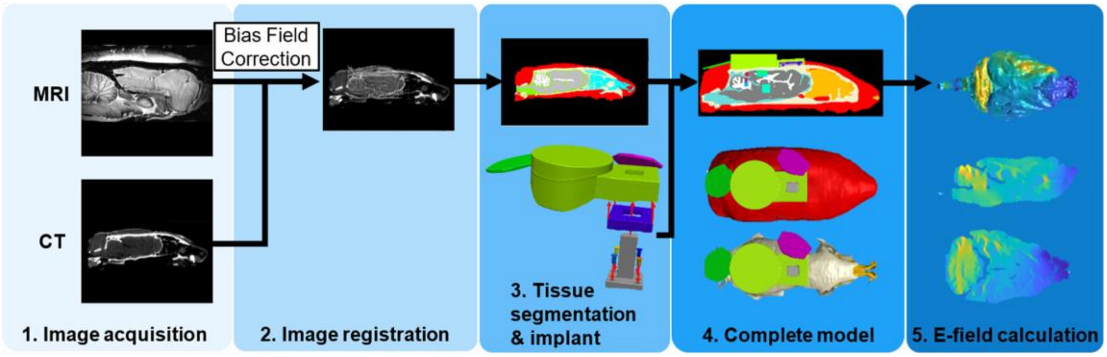

Supplementary Figure S5 Pipeline for computational modeling for tACS electric field in ferret head. The CT and MRI imaging data were co-registered to allow segmentation of hard and soft tissues (1-3). Implanted hardware and surface tACS electrodes were added to this segmentation (3) and meshed (4) with finite elements to create a three-dimensional representation of the experimental setup. Tissue and hardware conductivities were assigned based on the literature (see Supplementary Table S1) and COMSOL was used to solve the quasi-static Poisson equation for nodal electric potentials, from which the electric field was calculated (5). Related to Figure 4.

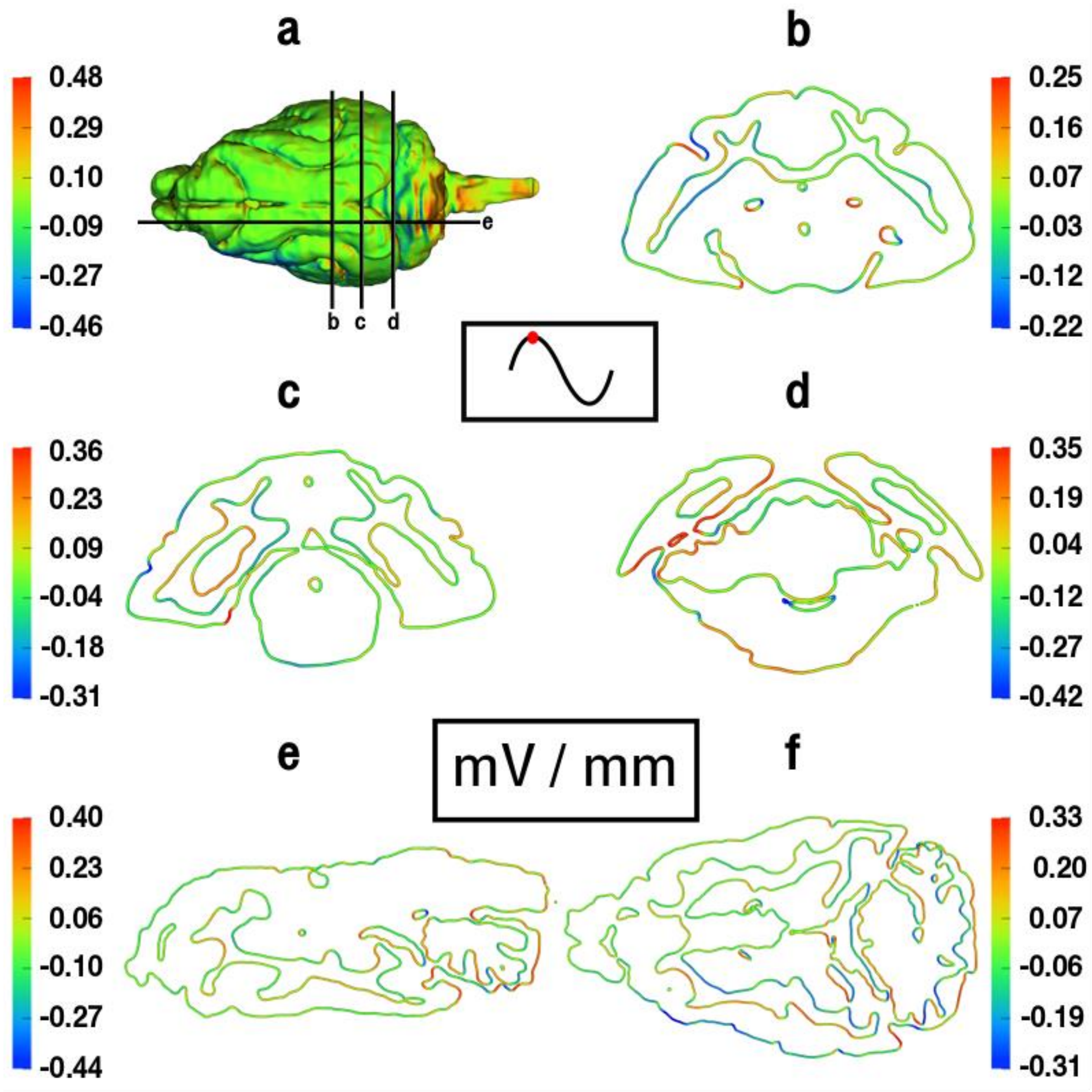

Supplementary Figure S6 Surface-normal component of electric field (nE) at 80 μA stimulation current depicted on: (a) ferret brain surface as well as through brain volume where nE is shown at gray matter interface only; (b-d) coronal slices, (e) sagittal and (f) axial brain slice. Related to Figure 4.

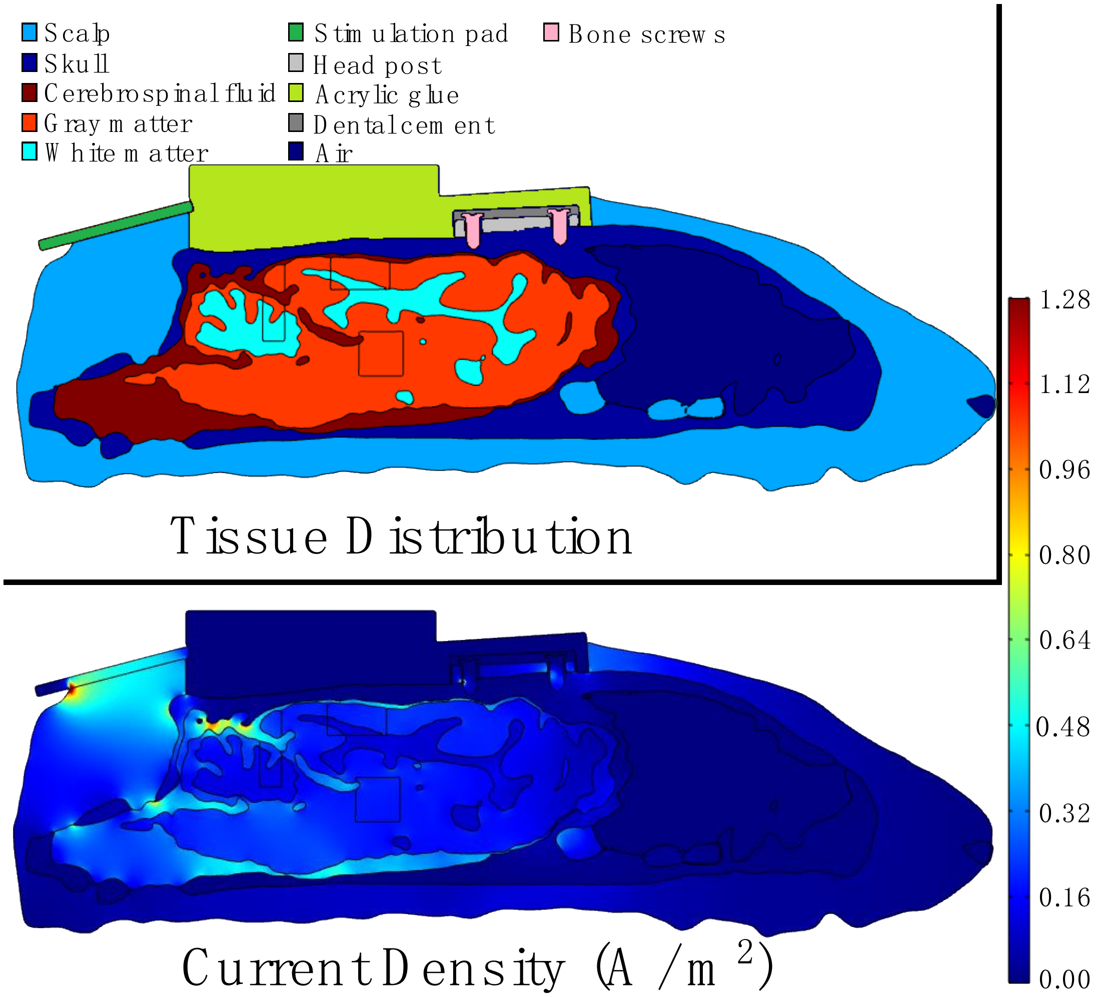

Supplementary Figure S7 Current density (80 µA) in sagittal cross-sectional view. Plane cuts through posterior tACS electrode and head post screws. Top: color-coded map of tissues and materials. Bottom: simulated current density shown here between 0–1.28 A/m^2 (volumetric maximum: 5.12 A/m^2 under electrode). The head screws perturb the current density only in their immediate vicinity near the skull–CSF interface. An advantageous feature of the experimental setup is the lack of significant current channeling through the screws, which is due to them being coated with insulating dental cement and acrylic glue on top. Related to Figure 4.

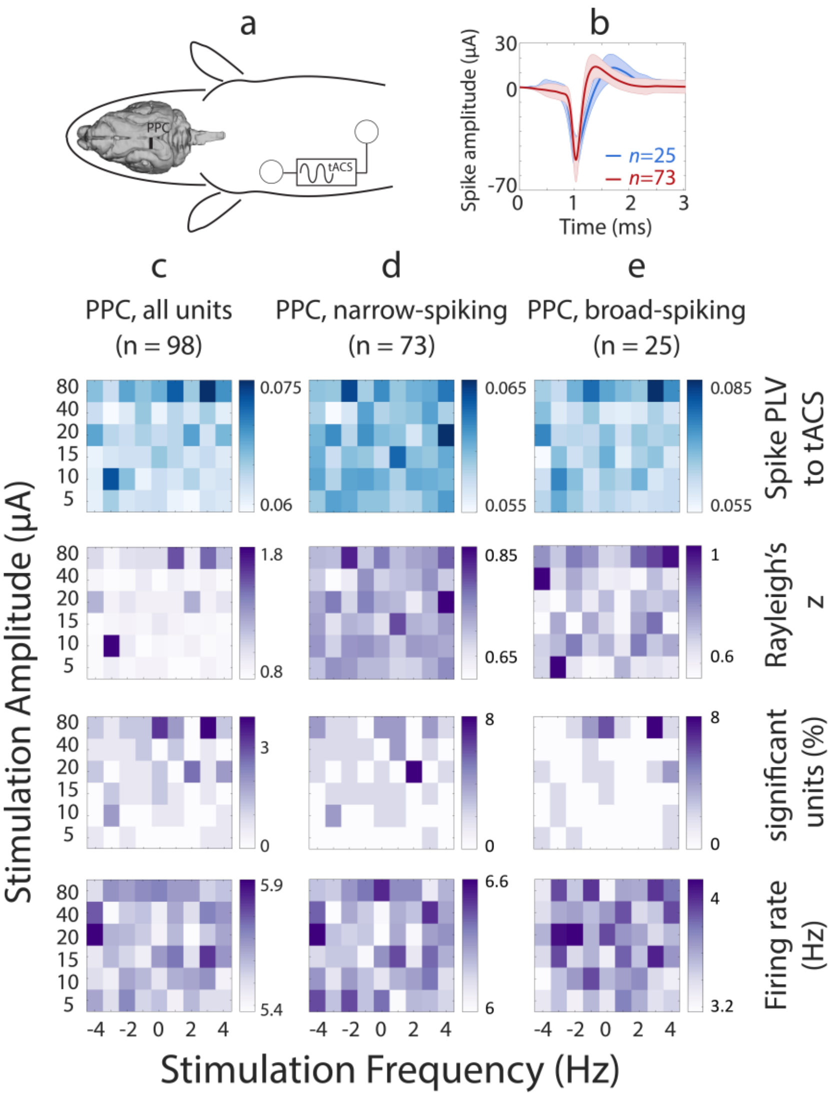

Supplementary Figure S8 Peripheral nerve stimulation failed to produce an Arnold tongue pattern in PPC at the level of individual neurons. Synchronization maps (blue, top row) and corresponding Rayleigh's z-score (purple, second row), percentage of units with significant phase-locking value (purple, third row) and firing rate maps (purple, bottom row) as a function of stimulation parameters for PPC. The synchronization maps show phase locking between individual spikes and the alternating current stimulation waveform as measured by phase-locking value (PLV) averaged across units. The horizontal axis indicates the distance (in Hz) from individual alpha frequency, and the vertical axis shows the stimulation amplitude.

a) Illustration of peripheral nerve stimulation by placement of the stimulation electrodes on the lower back of the ferret. The relative distance between the stimulation pads as well as stimulation parameters were all identical to those in the main tACS experiment in Figure 5.

b) Color coded spike waveforms (mean±SEM) for two identified clusters of narrow-spiking (red, n=73) and broad-spiking (blue, n=25) neurons in PPC.

(c-e, top two rows) There is no clear pattern of entrainment of PPC neurons centered at the individual endogenous alpha frequency.

(c-e, third row) A small percent of units (<8%) show significant modulation of spike phases as measured by Rayleigh's test for both types of neurons, however, due to the small sample size, the testing is highly unreliable.

(c-e, bottom row) The random pattern of firing rate maps for all unit types indicates that stimulation of peripheral nerves did not modulate the firing rate of the target neurons.

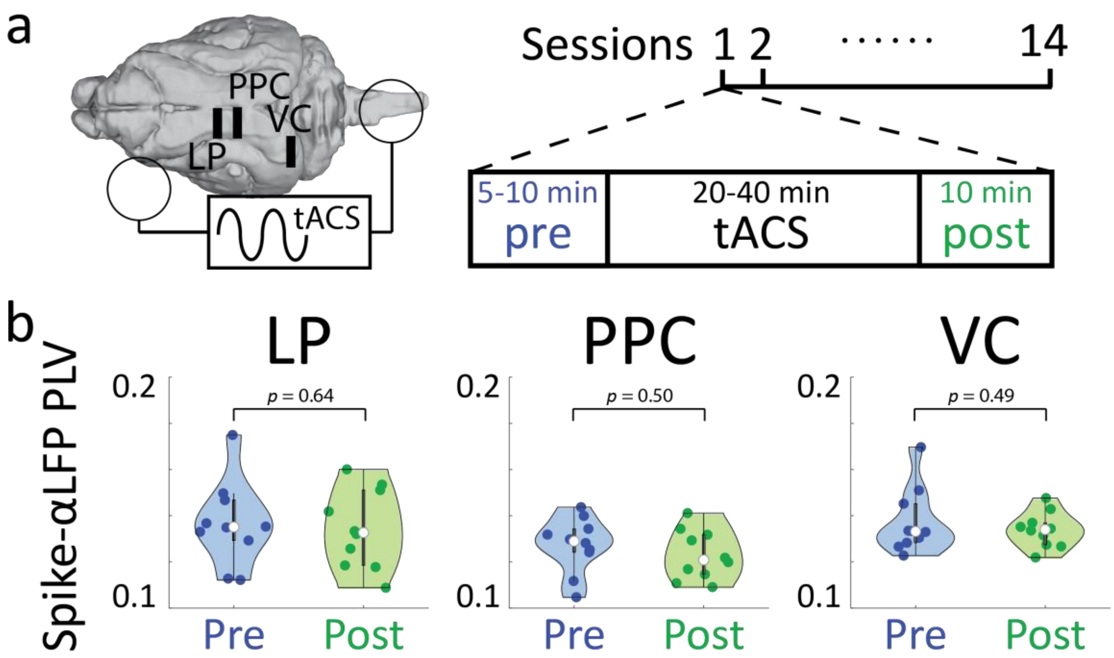

Supplementary Figure S9 Repeated tACS sessions do not produce long-lasting effect on neuronal synchrony. a) Illustration of the experimental setup. The tACS pads were placed at the same location as the tACS experiment for determination of the Arnold tongue. b) Scatter plots showing phase-locking value between single units and the endogenous alpha oscillation for pre (blue) and post (green) each tACS session. The violin plots showed the kernel density of PLV for each condition. The white middle point represented the median of data, the thick line flanking the middle point is the interquartile range. Each dot represents one PLV value between one single unit and alpha frequency component of the average LFP. The differences in phase-locking values between pre and post are not significant in all three regions (paired two-sided t-test, n=10 single units, LP: *p*=0.64, PPC: *p*=0.50, VC: *p*=0.49, without multiple comparisons adjustment), indicating the absence of a long-lasting effect after prolonged tACS sessions (20-40 minutes). Source data are provided as a Source Data file.

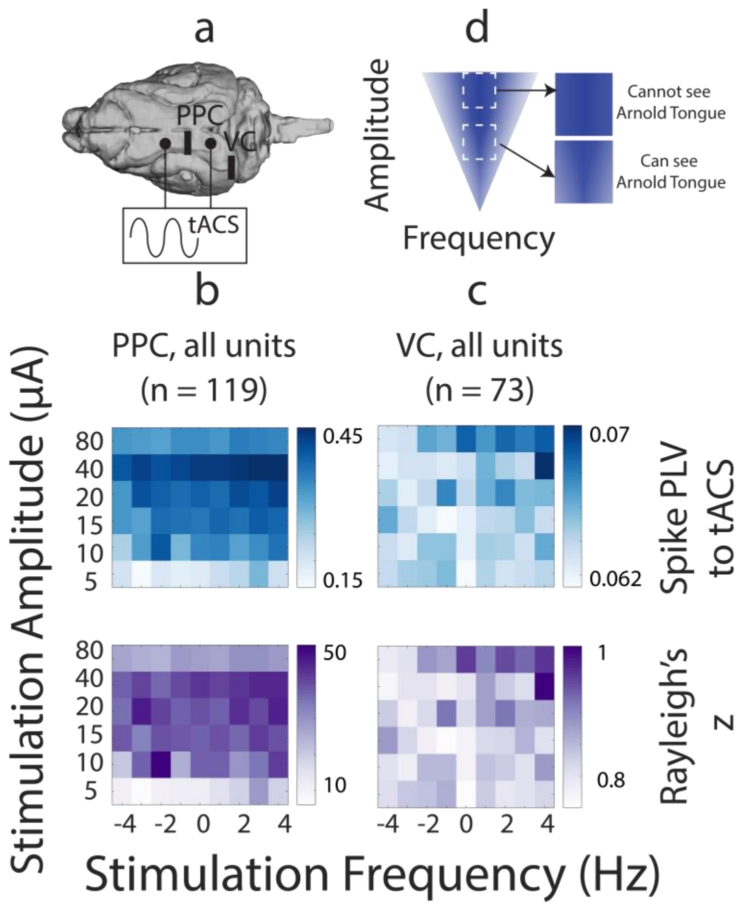

Supplementary Figure S10 Local bone screw stimulation engages cortical alpha oscillations at the level of individual neurons. Synchronization maps (blue) and corresponding Rayleigh's z-score (purple, second row) as a function of stimulation parameters for PPC and VC. The synchronization maps show phase locking between individual spikes and the stimulation waveform as measured by phase-locking value (PLV) averaged across units. The horizontal axis indicates the distance (in Hz) from individual alpha frequency, and the vertical axis shows the stimulation amplitude.

a) Illustration of bone screw stimulation near PPC in the anterior-posterior axis.

b) There is a gradual increase in PPC neuron entrainment as the amplitude increases from 10 to 40 µA. There is no clear pattern of entrainment of PPC neuron centered at the individual endogenous alpha frequency.

c) The synchronization in VC shows a preference to higher amplitude and higher frequency (relative to the endogenous frequency), but no clear preference to the individual endogenous alpha frequency for lower stimulation amplitudes.

d) A cartoon illustration of the Arnold Tongue shows the expected synchronization behavior for different parameter subspaces. When the amplitude is higher, such as in our bone screw stimulation experiment, the synchronization map is saturated and it is hard to see an Arnold Tongue pattern (top). When the amplitude is in the lower range, such as in our tACS experiment, the synchronization map shows a clearer Arnold Tongue pattern (bottom).

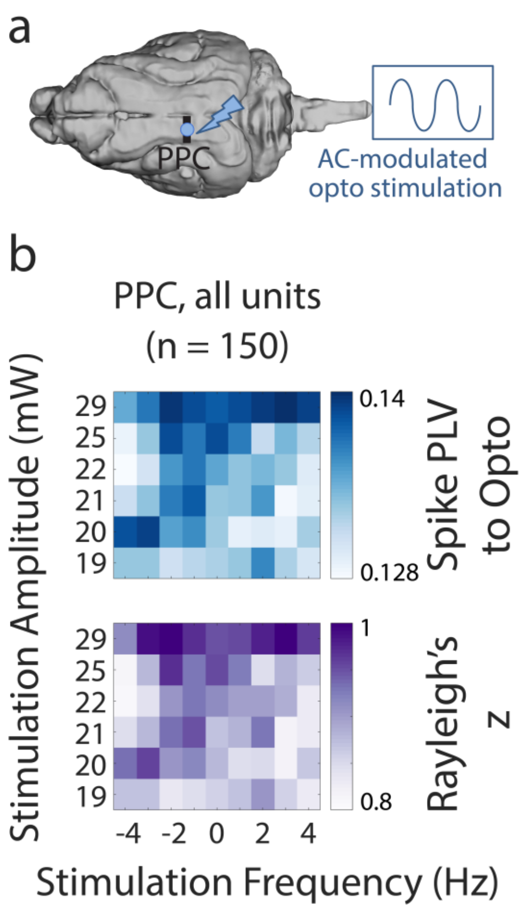

Supplementary Figure S11 Optogenetic stimulation in PPC engages cortical alpha oscillations at the level of individual neurons. Synchronization maps (blue) and corresponding Rayleigh's z-score (purple, second row) as a function of stimulation parameters for PPC and VC. The synchronization maps show phase locking between individual spikes and the stimulation waveform as measured by phase-locking value (PLV) averaged across units. The horizontal axis indicates the distance (in Hz) from the individual alpha frequency, and the vertical axis shows the stimulation amplitude.

a) Illustration of optogenetics stimulation in PPC with simultaneous electrophysiology recording. Laser power was modulated by a sinusoidal wave to mimic ACS.

b) Top: Synchronization plot between PPC units and the laser amplitude modulation displayed an Arnold tongue shape. The highest amplitude (29 mW) entrained neuron at all frequencies tested, and the lowest amplitude (19 mW) also did not show preference for a specific frequency. However, in between the two extremes, we observed amplitude- and frequency-dependent synchronization around the endogenous frequency, albeit a little left shifted. Bottom: The z-statistics from Rayleigh's test for non-uniformity of circular data also displays a similar triangular shape for PPC neurons, confirming the amplitude- and frequency-dependent spike-phase modulation.

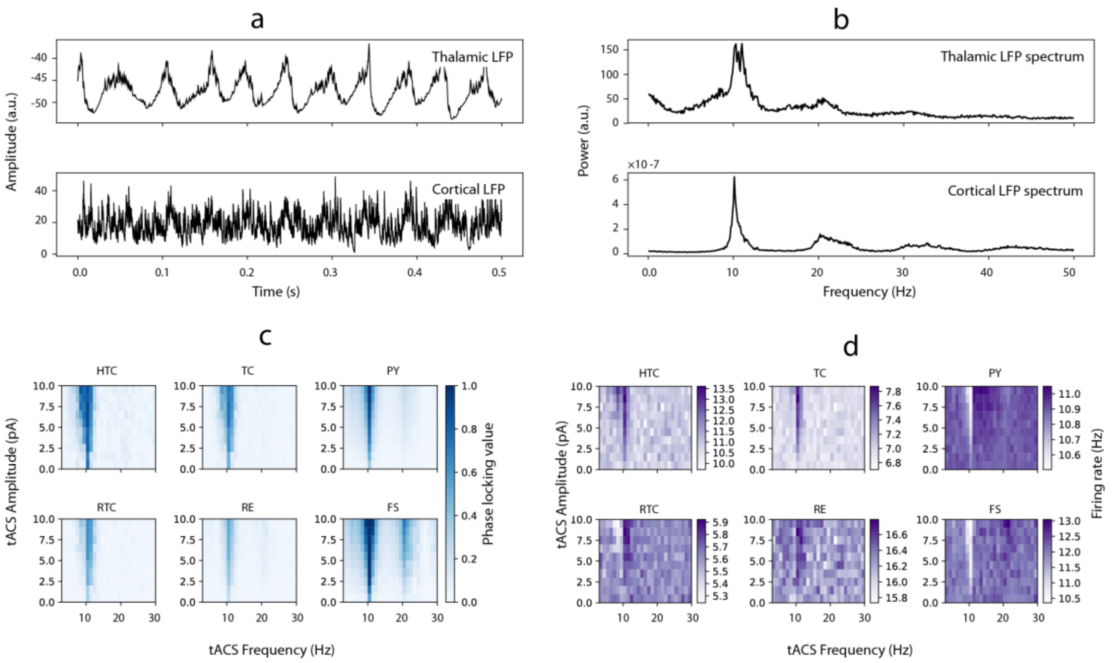

Supplementary Figure S12 Synchronization by tACS in modified thalamo-cortical network. In this modified network, both thalamus and cortex exhibit a spectral peak in the alpha frequency peak. In contrast to the main model (and the experimental data), thalamus does not exhibit a theta spectral peak anymore. As a result, tACS in the alpha frequency entrains both the cortical and thalamic neuronal populations in this modified model. HTC: excitatory high-threshold bursting thalamic cells, TC: thalamo-cortical cells, PY: excitatory pyramidal neurons, RTC: thalamo-cortical relay cells, RE: inhibitory thalamic reticular cells, FS: fast-spiking inhibitory neurons

a) Both cortical and thalamic LFPs show alpha endogenous oscillations. Raw traces of simulated LFPs.

b) Spectra for LPF traces shown in a).

c) Color-coded phase locking value (PLV) of spiking activity of different neuron types to tACS as a function of tACS frequency (horizontal axis) and tACS amplitude (vertical axis). Triangular-shape high-synchrony Arnold tongue regions centered on alpha frequency are evident for all neuron types.

d) Color-coded firing rate maps corresponding to PLV maps for all neuron types.

Related to Figure 6.

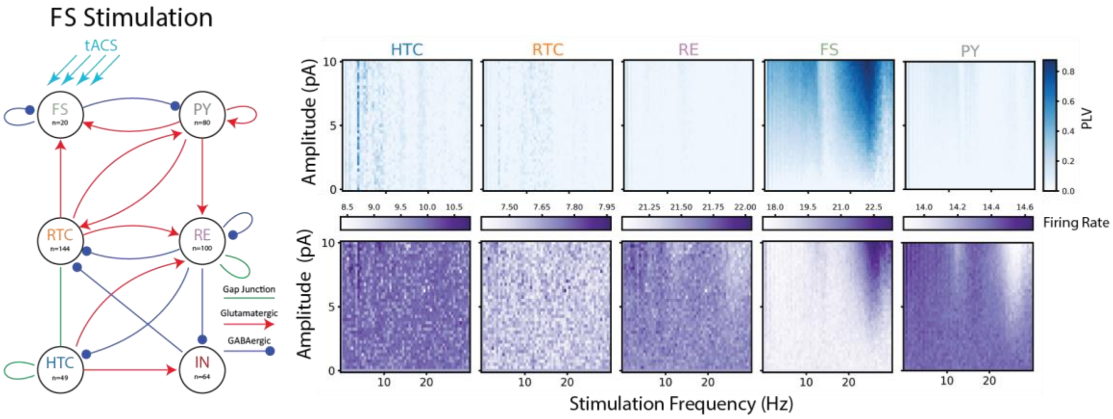

Supplementary Figure S13: Modeling tACS Stimulation to FS Neurons Only. Thalamocortical model response to different amplitudes and frequencies of tACS s applied only to FS neurons. Each pixel in the histogram represents five seconds of simulation with the appropriate amplitude and frequency tACS wave. tACS amplitude ranges from 0–10 pA and tACS frequency ranges from 0–30 Hz. Arnold tongues appear at the same frequencies as they do when both PY and FS cells receive tACS stimulation (Figure 7). Phase locking in the FS neurons is higher at the first harmonic compared to simulations where FS neurons do not receive stimulation.

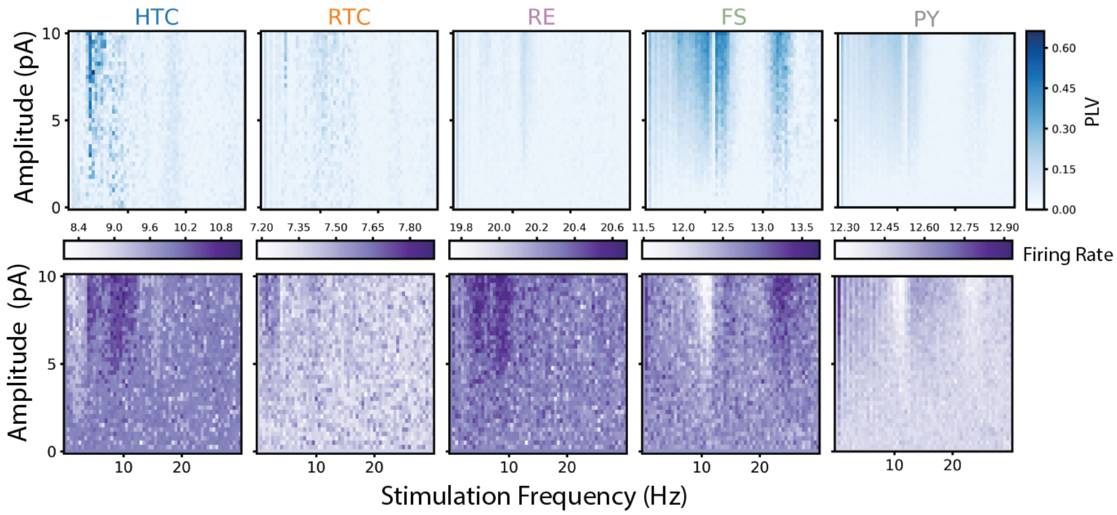

Supplementary Figure S14: Modeling tACS Stimulation in Model with NMDA receptors in Cortical Network. When NMDA synapses are added to the cortical portion of the model and biases are adjusted to achieve similar firing rates, the model exhibits similar phase locking and firing rate behavior to the model without cortical NMDA synapses.

Supplementary Table S1 Tissue/Material electrical conductivities. Related to Figure 4.

Tissue/Material	Electrical Conductivity [S/m]
Scalp	0.29
Skull	0.03
Cerebrospinal fluid	1.65
Grey matter	0.82
White matter	0.38
Stimulation pad	5.9×10^{7}
Head post, bone screws	1.39×10^{6}
Acrylic glue	3.6×10^{-5}
Dental cement	2.5×10^{-14}
Air	2.5×10^{-14}
