# Supplementary material for: Transcranial alternating current stimulation entrains alpha oscillations by preferential phase synchronization of fast-spiking cortical neurons to stimulation waveform
Source: Nat Commun. 2021 May 25;12:3151. doi: 10.1038/s41467-021-23021-2 (PMC8149416; doi:10.1038/s41467-021-23021-2)
Supplement: Supplementary file 2 — Reporting Summary [file 41467_2021_23021_MOESM2_ESM.pdf]

nature research

Corresponding author(s): Flavio Frohlich

Last updated by author(s): Feb 13, 2021

Reporting Summary

Nature Research wishes to improve the reproducibility of the work that we publish. This form provides structure for consistency and transparency in reporting. For further information on Nature Research policies, see our Editorial Policies and the Editorial Policy Checklist.

Statistics

For all statistical analyses, confirm that the following items are present in the figure legend, table legend, main text, or Methods section.

n/a	Confirmed	
[ ]	[x]	The exact sample size (n) for each experimental group/condition, given as a discrete number and unit of measurement
[ ]	[x]	A statement on whether measurements were taken from distinct samples or whether the same sample was measured repeatedly
[ ]	[x]	The statistical test(s) used AND whether they are one- or two-sided<br>*Only common tests should be described solely by name; describe more complex techniques in the Methods section.*
[x]	[ ]	A description of all covariates tested
[ ]	[x]	A description of any assumptions or corrections, such as tests of normality and adjustment for multiple comparisons
[ ]	[x]	A full description of the statistical parameters including central tendency (e.g. means) or other basic estimates (e.g. regression coefficient) AND variation (e.g. standard deviation) or associated estimates of uncertainty (e.g. confidence intervals)
[ ]	[x]	For null hypothesis testing, the test statistic (e.g. F, t, r) with confidence intervals, effect sizes, degrees of freedom and P value noted<br>*Give P values as exact values whenever suitable.*
[x]	[ ]	For Bayesian analysis, information on the choice of priors and Markov chain Monte Carlo settings
[x]	[ ]	For hierarchical and complex designs, identification of the appropriate level for tests and full reporting of outcomes
[x]	[ ]	Estimates of effect sizes (e.g. Cohen's d, Pearson's r), indicating how they were calculated

Our web collection on statistics for biologists contains articles on many of the points above.

Software and code

Policy information about availability of computer code

Data collection	Electrophysiological data was collected using Matlab R2016b. Ferret head model data was collected using 3D slicer 4.8.0, SimpleWare ScanIP O-2018.12, and SolidWorks 2016 X64 Edition.
Data analysis	We used Kilosort2 for spike sorting, COMSOL Multiphysics 5.4/5.5 for E-field / visualization, MATLAB 2019a and SCIRun 4.7 (R45839) for potential measurement, E-field and current density analysis / visualization. All other analysis methods were implemented and performed in Matlab (R2016b).

For manuscripts utilizing custom algorithms or software that are central to the research but not yet described in published literature, software must be made available to editors and reviewers. We strongly encourage code deposition in a community repository (e.g. GitHub). See the Nature Research guidelines for submitting code & software for further information.

Data

Policy information about availability of data

All manuscripts must include a data availability statement. This statement should provide the following information, where applicable:

- Accession codes, unique identifiers, or web links for publicly available datasets
- A list of figures that have associated raw data
- A description of any restrictions on data availability

Electrophysiological data can be made available from the corresponding author upon request.

Field-specific reporting

Please select the one below that is the best fit for your research. If you are not sure, read the appropriate sections before making your selection.

- [x] Life sciences
- [ ] Behavioural & social sciences
- [ ] Ecological, evolutionary & environmental sciences

For a reference copy of the document with all sections, see nature.com/documents/nr-reporting-summary-flat.pdf

Life sciences study design

All studies must disclose on these points even when the disclosure is negative.

Sample size	No sample size calculation was performed. 3 animals were used for the main experiments, with n=l6, 21, 14 sessions for animals 1 to 3 respectively. The number of sessions were determined based on the availability of spike data from implanted electrodes at each region of interest, which was about 3-4 weeks after implant surgery. No recording was performed in the first week after surgery, leaving a 2-3 week time-window of access to reliable spike signals. Additional control experiments were performed, with 2 animals for peripheral nerve stimulation, 1 animal for longer duration stimulation sessions, 3 animals for bone screw stimulation, and 2 animals for optogenetic stimulation. These samples are sufficient due to the highly similar results from the three animals. Like studies in non-human primates, studies of new ideas often start small and the marginal return diminishes with increasing sample size (Bacchetti, 2011).
Data exclusions	We included data from all animals.
Replication	We do not include any specific measure to verify the reproducibility of the experimental findings. Each experiment was done in multiple animals in one setting. No direct replication was performed.
Randomization	We randomized the order in which the tACS with different stimulation parameters was applied within each session.
Blinding	Blinding was not relevant to this study since it is an in vivo animal study, and no behavioral or task is performed by animals. Because all randomized conditions (i.e. different stimulation parameters) in each session are embedded in a continuous electrophysiological recording which was processed all together regardless of the condition. There is no way to bias the data or result towards one condition or another.

Reporting for specific materials, systems and methods

We require information from authors about some types of materials, experimental systems and methods used in many studies. Here, indicate whether each material, system or method listed is relevant to your study. If you are not sure if a list item applies to your research, read the appropriate section before selecting a response.

Materials & experimental systems

n/a	Involved in the study	
x		Antibodies
x		Eukaryotic cell lines
x		Palaeontology and archaeology
	x	Animals and other organisms
x		Human research participants
x		Clinical data
x		Dual use research of concern

Methods

n/a	Involved in the study	
x		ChIP-seq
x		Flow cytometry
x		MRI-based neuroimaging

Animals and other organisms

Policy information about studies involving animals; ARRIVE guidelines recommended for reporting animal research

Laboratory animals	Adult spayed female ferrets (Mustela putorius furo) were used in this study. The animals were 4 month old at the beginning of the experiment.
Wild animals	This study did not involve wild animals.
Field-collected samples	This study did not involve sa mp l es collected from field animals.
Ethics oversight	Institutional Animal Care and Use Committee of the University of North Carolina at Chapel Hill

Note that full information on the approval of the study protocol must also be provided in the manuscript.
